# Modeling disordered protein interactions from biophysical principles

**DOI:** 10.1371/journal.pcbi.1005485

**Published:** 2017-04-10

**Authors:** Lenna X. Peterson, Amitava Roy, Charles Christoffer, Genki Terashi, Daisuke Kihara

**Affiliations:** 1 Department of Biological Sciences, Purdue University, West Lafayette, Indiana, United States of America; 2 Department of Medicinal Chemistry and Molecular Pharmacology, Purdue University, West Lafayette, Indiana, United States of America; 3 Bioinformatics and Computational Biosciences Branch, Rocky Mountain Laboratories, NIAID, National Institutes of Health, Hamilton, Montana, United States of America; 4 Department of Computer Science, Purdue University, West Lafayette, Indiana, United States of America; 5 School of Pharmacy, Kitasato University, Tokyo, Japan; Center for Cancer Research, UNITED KINGDOM

## Abstract

Disordered protein-protein interactions (PPIs), those involving a folded protein and an intrinsically disordered protein (IDP), are prevalent in the cell, including important signaling and regulatory pathways. IDPs do not adopt a single dominant structure in isolation but often become ordered upon binding. To aid understanding of the molecular mechanisms of disordered PPIs, it is crucial to obtain the tertiary structure of the PPIs. However, experimental methods have difficulty in solving disordered PPIs and existing protein-protein and protein-peptide docking methods are not able to model them. Here we present a novel computational method, IDP-LZerD, which models the conformation of a disordered PPI by considering the biophysical binding mechanism of an IDP to a structured protein, whereby a local segment of the IDP initiates the interaction and subsequently the remaining IDP regions explore and coalesce around the initial binding site. On a dataset of 22 disordered PPIs with IDPs up to 69 amino acids, successful predictions were made for 21 bound and 18 unbound receptors. The successful modeling provides additional support for biophysical principles. Moreover, the new technique significantly expands the capability of protein structure modeling and provides crucial insights into the molecular mechanisms of disordered PPIs.

## Author summary

A substantial fraction of the proteins encoded in genomes are intrinsically disordered proteins (IDPs), which lack a single stable structure in the native state. IDPs serve many functions including mediating protein-protein interactions (PPIs). Such disordered PPIs are prevalent in important regulatory pathways, including many interactions of the tumor suppressor protein p53. To elucidate the molecular mechanisms of disordered PPIs, obtaining tertiary structure information is essential; however, they are difficult to study with experimental techniques and existing computational protein-protein and protein-peptide modeling methods are unable to model disordered PPIs. Here we present a novel computational method for modeling the structure of disordered PPIs, which is the first of this sort. The method, IDP-LZerD, is designed to follow a known biophysical picture of the mechanism of how IDPs interact with structured proteins. IDP-LZerD successfully modeled the majority of disordered PPIs tested. This technique opens up new possibilities for structural studies of IDPs and their interactions.

## Introduction

Intrinsically disordered proteins (IDP), which have evolved to not adopt a stable structure under physiological conditions, are a departure from the traditional paradigm of structured proteins [1]. After initial recognition of their critical biological functions in the 1990s [1], IDPs quickly gained attention as they were found to be abundant in genomes across all three kingdoms [2]. IDPs are known to be involved in many molecular recognition events. Particularly, it is estimated that 15–45% of protein-protein interactions (PPIs) are formed with IDPs [3]. A well-known example is the p53 tumor suppressor, which contains disordered regions that interact with dozens of partner proteins [4]. Due to the abundance and characteristic features of IDPs in PPI networks, including many critical signaling pathways, fully understanding the molecular mechanisms of PPI networks requires consideration of the role of interactions with IDPs.

The binding mechanism of an IDP to a structured target protein, i.e. a disordered PPI, has drawn much interest in the context of binding rate constants, because disordered PPIs achieve high specificity and high dissociation rate constant simultaneously, which is an ideal characteristic for signaling pathways but difficult to realize with interactions of structured proteins [5]. It is generally accepted that binding precedes global folding of the IDP, although secondary structures in local regions may form before the interaction. In the model called the dock-and-coalesce [5], a small segment of the IDP, which may be folded into secondary structure prior to binding, forms the initial contact with the ordered partner, followed by coalescence of the rest of the IDP into the bound conformation. This mechanism imparts both thermodynamic and kinetic advantages. Forming a binding interface out of segments leads to a large interface with fewer amino acids than a structured protein [2, 6] and the binding affinity is accumulated from the affinities of each segment [5]. This allows IDPs to have high binding specificity, but the loss of entropy upon binding imparted by the flexibility makes the interaction reversible [7]. From a kinetic perspective, sequential binding of individual segments will have a much higher rate constant than a hypothetical situation in which a pre-organized IDP simultaneously makes all contacts with the ordered protein [5]. A computational method based on the dock-and-coalesce model was successful in predicting the binding rate constants of disordered PPIs [8].

Experimental structure determination of disordered PPIs using techniques such as X-ray crystallography and nuclear magnetic resonance (NMR) is challenging due to the flexible nature of IDPs and their tendency to form weak, transient interactions [9]. Indeed, not all IDPs form a single, stable structure when bound. Examples of these so-called “fuzzy” complexes are cataloged in FuzDB [10, 11]. Along a similar line, pE-DB contains ensembles of conformations that can be adopted by an IDP [12]. Nevertheless, many proteins annotated as disordered in DisProt [13] do adopt a bound structure that can be experimentally determined.

For PPIs of structured proteins, experimental structure methods can often be complemented by computational modeling of protein complexes (docking) [14]. However, current rigid-body and flexible docking methods (which allow small conformational changes at the docking interface) are not able to model disordered PPI prediction, because the required rigid structures are not available for IDPs. Among existing protein modeling techniques, peptide-protein docking methods would be the most similar to disordered PPI prediction. Approaches to peptide-protein complex modeling include template-based modeling (TBM) [15, 16], molecular dynamics (MD) [17–19], small molecule docking [20, 21], protein-protein docking with flexibility [22–26], and coarse-grained docking [27]. The characteristics of the docking and MD methods are compared in Table 1. Several of the methods require knowledge of the binding site as input. Information about the binding site can be obtained experimentally or by using computational prediction of peptide binding sites [28–30] or protein binding sites [31, 32]. More fundamentally, existing methods were developed and tested for binding short peptides of 2–16 residues, which is far shorter than the 10–70 residue IDPs that participate in disordered PPIs [2], although some programs are able to accept peptides up to 30 residues in their web servers. To predict the tertiary structure of a disordered PPI, a method must solve two interdependent problems: the tertiary structure of the input sequence of the disordered protein and its binding location on the receptor protein. This is a difficult task as the conformational space to be explored for an IDP is enormous and grows with its length. Currently, no existing methods can dock a long disordered protein to its receptor protein. A totally new approach is required for predicting the structure of a disordered PPI involving commonly observed long IDPs.

**Table 1:** Existing peptide-protein complex modeling methods.

Method	Category	Availability	Requires binding site	Initial peptide conformation	Tested (max) amino acids^a^
*Hetenyi et al*. [20]	Docking	No	No	TINKER [33]	4
*Liu et al*. [22]	Docking	No	Yes	Bound conformation	16
Rosetta FlexPepDock ab-initio [23, 24]	Docking	Yes	Yes	Predicted fragments	15 (30)
HADDOCK [25]	Docking	Yes	Yes	*α*-helix, extended, polyproline	15
pepATTRACT [26]	Docking	Yes	No	*α*-helix, extended, polyproline	15
CABS-DOCK [27]	Docking	Yes	No	Random	15 (30)
MdockPeP [21]	Docking	No	No	Sequence-based search	15
DynaDock [17]	MD	No	Yes	Bound conformation	16
*Dagliyan et al*. [18]	MD	No	No	Bound conformation	13
AnchorDock [19]	MD	No	No	Extended/MD	15

^a^: Tested is the longest peptide in the published test set and max is the maximum length allowed by the web server.

In this work, we describe the development of a novel computational method named IDP-LZerD, which is able to model for the first time the docked structure of long IDPs (15–69 amino acids). IDP-LZerD applies the biophysical principles of the dock-and-coalesce mechanism of IDP binding to model the structures of long IDPs. In the “dock” phase, small segments of the IDP are modeled in various conformations and docked globally to the ordered protein. Modeling and docking small segments is not only faster and easier but also consistent with the biophysical mechanism of small segments of the IDP binding sequentially. In the “coalesce” phase, the docked segments of neighboring regions of the IDP are found and combined into a complete structure of the disordered PPI. We found that correct bound conformations of the IDP were selected using scores evaluating docking with the receptor, which corresponds to the biophysical model that the conformation of an IDP is stabilized and determined by contacts with its receptor. In addition, the combination of the docking scores of multiple segments is analogous to the accumulation of the binding affinities of multiple segments [5]. Overall, we show that IDP-LZerD is able to yield docking models of a practical quality in a number of bound and unbound structures of PPIs involving long IDPs.

## Results

**Fig 1:**
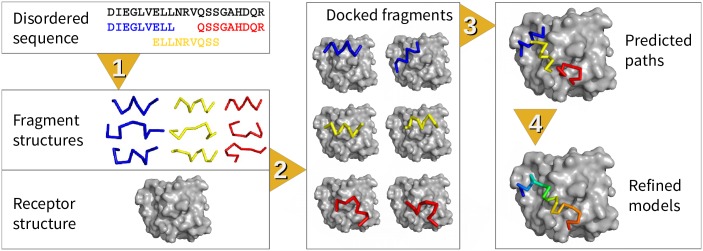
IDP-LZerD consists of four steps. 1. fragment structure prediction, 2. fragment docking, 3. path assembly, and 4. refinement. Steps 1 and 2 correspond to “dock” and Steps 3 and 4 correspond to “coalesce.”

The steps of IDP-LZerD are outlined in Fig 1. The binding mechanism of a disordered PPI is well described by a dock-and-coalesce model, in which a small segment of the IDP makes initial contact with the ordered protein, forming a seed for the rest of the IDP to explore the conformational space and coalesce into the final bound conformation [5]. In a disordered PPI, the interface of the IDP is typically formed by one or a few continuous segments [6], and the bound structure of the IDP is often correctly predicted using sequence-based secondary structure prediction [34]. Based on these biological insights, conformations for 9-residue sequence windows of the IDP sequence are predicted from their sequences and their predicted secondary structure (Step 1). These fragment structures are then docked to a receptor with a rigid-body protein-protein docking method, LZerD [35–37] (Step 2). Finally, the docked fragments are assembled into a full-length IDP complex, called a path (Step 3), and refined to construct final models of the disordered PPI (Step 4). Steps 1 and 2 correspond to the “dock” phase of dock-and-coalesce, finding potential seed contacts between an IDP segment and the ordered protein, while Steps 3 and 4 correspond to “coalesce”, the formation of all of the contacts that stabilize the complex between the IDP and the ordered protein. In this manner, the challenging problem of simultaneously predicting the IDP structure and its binding conformation is divided into feasible sub-problems. The method was trained on fourteen complexes (Table 2) and tested on eight complexes (Table 3). For all complexes, both bound and unbound receptor structures available in the PDB [38] were used. A bound case is where a target IDP is docked to the IDP-bound form of a receptor protein while in an unbound case the IDP is docked to a structure of a receptor that was determined without its ligand IDP. Due to conformational changes upon binding, predicting the docking pose using an unbound receptor protein is in general more difficult. The training set was used to train weights of scoring functions and evaluate the performance at each step while the test set was used to evaluate the performance at the end. Accuracy is measured using the CAPRI criteria of *f*_*nat*_, I-RMSD, and L-RMSD, detailed in S1 Table [39]. *f*_*nat*_ is the fraction of native residue-residue contacts shown in the model, I-RMSD is the root mean square deviation (RMSD) of the interface residues, and L-RMSD is the RMSD of the bound ligand after superimposition using the receptor. Each step and results are further discussed below.

**Table 2:** Disordered protein complex data set.

Disordered protein name	Receptor protein name	Bound			Unbound		DisProt ID or ref.
		Receptor PDB ID	Ligand chain	L	Receptor PDB ID	Pocket RMSD (Å)	
P53, transactivation domain	MDM2, N-terminal domain	1ycrA	B	15	1z1mA^a^	2.93	DP00086
Myelin basic protein	MHC class II antigen DRA/DRB5	1fv1AB	C	20	4ah2AB^b^	0.91	DP00237
eIF4E-binding protein 1	eukaryotic initiation factor 4E	1wkwA	B	20	1ipbA	0.78	DP00028
Protein kinase inhibitor *α*	PKA C-*α*	2cpkE	I	20	1j3hA	4.57	DP00015
c-Myb	Cbp/p300, KIX domain	1sb0A	B	25	4i9oA^c^	2.80	[40]
Cibulot	*α*-actin-1	1sqkA	B	25	1ijjA	0.79	[41]
Bcl2-associated Antagonist of cell Death (BAD)	Bcl2-like protein 1 (Bcl2-L-1)	2bzwA	B	27	1pq0A	3.00	DP00563
Regulatory protein SIR3	DNA-binding protein RAP1	3owtAB	C	27	3cz6AB^d^	1.30	DP00533
hSARA, SMAD2-binding domain	hSMAD2	1devA	B	41	1khxA	3.94	DP00141
Cbp/p300-interacting transactivator 2 (CITED2)	Cbp/p300, TAZ1 domain	1p4qB^e^	A	44	1l3eB^e^	5.11	DP00356
Transcription factor 7-like 2 (TCF7L2)	*β*-catenin	1jpwA	D	45	2z6hA	0.98	DP00175
Hypoxia-inducible factor 1-*α* (Hif-1*α*)	Cbp/p300, TAZ1 domain	1l8cA	B	51	1u2nA	2.87	DP00262
Nucleoporin NUP2	Importin subunit *α*	2c1tA	C	51	1bk5A^f^	1.44	DP00222
Synaptosomal-associated protein 25, SNARE domain	Botulinum neurotoxin type A (BoNT/A)	1xtgA	B	59	1xtfA	4.24	DP00068

^a^: removed residues 1 to 24;

^b^: removed chain B engineered residues -30 to 0;

^c^: removed the stabilizing small molecule KI1 (1-4-[4-chloro-3-(trifluoromethyl)phenyl]-4-hydroxypiperidin-1-yl-3-sulfanylpropan-1-one);

^d^: superimposed 2 copies of 3cz6A onto 3owtAB;

^e^: both chains A and B of 1p4q are disordered, so to create an unbound receptor for 1p4qA from 1l3eBA, we removed chain A, which has a different sequence than 1p4qA;

^f^: removed homodimer.

**Table 3:** Disordered protein complex test set.

Disordered protein name	Receptor protein name	Bound			Unbound		DisProt ID or ref.
		Receptor PDB ID	Ligand chain	L	Receptor PDB ID	Pocket RMSD (Å)	
Peroxisomal targeting signal 1 receptor	PEX14	2w84A	B	20	5aonA^a^	1.19	DP00472
CDK inhibitor 1	Proliferating cell nuclear antigen	1axcA	B	22	1vymA	1.86	DP00016
Alpha trans-inducing protein	Transcriptional coactivator PC4	2pheAB	C	26	1pcfAB	1.87	DP00087
Protease A inhibitor 3	Proteinase A	1g0vA	B	31	1fmxA	3.80	DP00179
Nuclear factor erythroid 2-related factor 2	Keap1	3wn7A	B	35	1x2jA	0.90	DP00968
Protein phosphatase 1 regulatory subunit 12A	PP-1B	1s70A	B	39	4ut2A	0.92	DP00218
Protein phosphatase inhibitor 2	PP-1G	2o8gA	I	40	1jk7A	1.45	DP00815
Outer membrane virulence protein YopE	YopE chaperone SycE	1l2wAB	I	69	1jyaAB	1.27	[42]

^a^: template-based model using MODELLER [43] (5aonA was used as the template, which has 46.9% sequence identity to 2w84A).

### Secondary structure prediction

Secondary structure was predicted for each IDP using JPRED [44], Porter [45], SSPro [46], and PSIPRED [47]. The secondary structure predictions were reasonably accurate (Table 4). If the predictions are considered correct when any of the four methods predicts the correct secondary structure, the accuracy is 86%. For 57% of residues, all four methods predicted the correct secondary structure. Even in the minority of cases where none of the methods predicted the correct secondary structure, fragments of all three secondary structure classes were created (described below in Methods).

**Table 4:** Secondary structure prediction accuracy.

Method	Accuracy
JPred	66.4%
Porter	81.2%
PSIPRED	69.7%
SSpro	75.4%
All	57.0%
Best	86.1%

Accuracy: percentage of all residues correctly predicted. Secondary structure classes were assigned using DSSP [48]. DSSP classes GHI are considered H, EB are considered E, and all others are considered C. All: all four methods predict the correct class. Best: at least one of the four methods predicts the correct class. Computed using 1ycrB, 1fv1C, 1wkwB, 2cpkI, 1sb0B, 1sqkB, 2bzwB, 3owtC, 1devB, 1l8cB, and 1xtgB.

### IDP fragment structure prediction

The full sequence of a target disordered protein was divided into 9-residue windows with a 3-residue overlap. Fragment structures of each window were predicted using Rosetta Fragment Picker (RFP) [49], which predicts structures based on the sequence profile [50] and predicted secondary structure [44–47]. RFP was configured to output 30 fragments for a window. Increasing the number of fragments chosen did not yield structures of a substantially lower root mean square deviation (RMSD) to the native structure (S1 Fig). Fragment structure was predicted reasonably accurately: on average the largest backbone RMSD of 30 conformations for a window was 1.8 Å for the training set, 1.6 Å for the test set, and 1.8 Å overall (S2 Table).

### Docking fragments to receptor

For a sequence window, each of the 30 fragment structures was docked with the receptor protein using LZerD [35–37]. LZerD is a shape-based, rigid-body docking method with the advantage of a soft representation of the surface shape of a protein that accounts for some conformational change upon binding. Docked fragment poses were clustered and the top 4,500 cluster centers were selected (see Methods). Ranking was performed using the sum of the Z-scores of two scoring functions, DFIRE [51] and ITScorePro [52], named DI score. DI score was shown to perform better in docking pose selection than the individual scores (S3 Table).

The docking accuracy of fragments is summarized in the “All docked” columns in S2 Table. For bound cases, on average the worst (largest) of the minimum L-RMSD from all the windows in a target was 3.7 Å and 4.1 Å for the training and the testing set, respectively. For unbound cases, the values were slightly worse, 4.4 Å and 4.3 Å for the training and the testing set, respectively. Fragment structure and docking accuracy was further tested on an additional independent test set of 11 cases of 9-residue IDP complexes found in the database of eukaryotic linear motifs (ELMs) [53] (Table 5). The results are shown in Table 6. The average fragment RMSD is 1.4 Å and the average minimum docked RMSD is 3.2 Å for both bound and unbound cases (Table 6), which are better than the results shown in S2 Table.

**Table 5:** 9-residue IDR complex test set selected from ELM.

Disordered protein name	Receptor protein name	Bound			Unbound	DisProt ID	ELM ID
		Receptor PDB ID	Ligand chain	First res	Receptor PDB ID		
Cyclin-dependent kinase inhibitor 1B	CDK2/Cyclin A	1jsuAB	C	25	2c5nAB	DP00018	-
				34		DP00018	-
				43		DP00018	-
				52		DP00018	ELMI000069
PIFtide	Protein kinase Akt-2	1o6lA	A	469	1gzkA	DP00304	ELMI001633
Glycogen synthase kinase-3 *β*	Protein kinase Akt-2	1o6lA	C	4	1gzkA	DP00385	-
Protein phosphatase 1 regulatory subunit 12A	PP-1B	1s70A	B	1	4ut2A	DP00218	-
				10		DP00218	ELMI002747
				22		DP00218	-
				31		DP00218	ELMI001397
Peroxisomal targeting signal 1 receptor	PEX14	2w84A	B	101	5aonA^a^	DP00472	ELMI002213

^a^: template-based model using MODELLER [43] (5aonA was used as the template, which has 46.9% sequence identity to 2w84A).

**Table 6:** Fragment modeling and docking accuracy for 9-residue IDR complexes from ELM.

Bound PDB ID	First res	Minimum RMSD (Å)			Unbound PDB ID	Minimum RMSD (Å)	
		Fragments	All docked	Selected docked		All docked	Selected docked
1jsuAB	25	1.8	3.5	3.5	2c5nAB	3.2	3.2
1jsuAB	34	1.4	2.8	9.5	2c5nAB	3.2	9.2
1jsuAB	43	0.5	1.6	1.9	2c5nAB	1.6	2.6
1jsuAB	52	0.6	1.6	3.1	2c5nAB	3.1	3.1
1o6lA	4	2.1	3.9	3.9	1gzkA	3.6	4.6
1o6lA	469	2.9	5.1	5.7	1gzkA	5.5	5.5
1s70A	1	1.3	3.5	9.1	4ut2A	3.5	8.3
1s70A	10	0.4	3.1	3.1	4ut2A	2.6	2.6
1s70A	22	2.4	3.6	7.5	4ut2A	3.3	5.8
1s70A	31	1.6	3.8	4.1	4ut2A	3.3	4.5
2w84A	101	0.4	2.8	2.8	5aonA	2.0	2.0
Average		1.4	3.2	4.9		3.2	4.7

First res.: The first amino acid position of the 9-residue long fragments in the protein. Fragments: minimum backbone RMSD of predicted fragments to native. All docked: minimum L-RMSD of all docked fragments (has a lower bound of the fragment RMSD). Selected docked: minimum L-RMSD of top 4,500 fragments by DI score (Z(DFIRE) + Z(ITScorePro)).

Selection of docked fragments was successful for most of the training set complexes, with an average RMSD of 5.4 Å for bound and 6.5 Å for unbound (“Selected docked” columns in S2 Table). On the testing set, the results are similar, 5.7 Å and 6.3 Å for bound and unbound cases. Exceptions included 2clt and 1bk5, where poor selection of docked fragments prevented successful modeling in the subsequent steps. On the additional ELM-derived dataset, results were 4.9 Å for bound and 4.7 Å for unbound (Table 6), which are again comparable to the results on the testing and training datasets.

**Fig 2:**
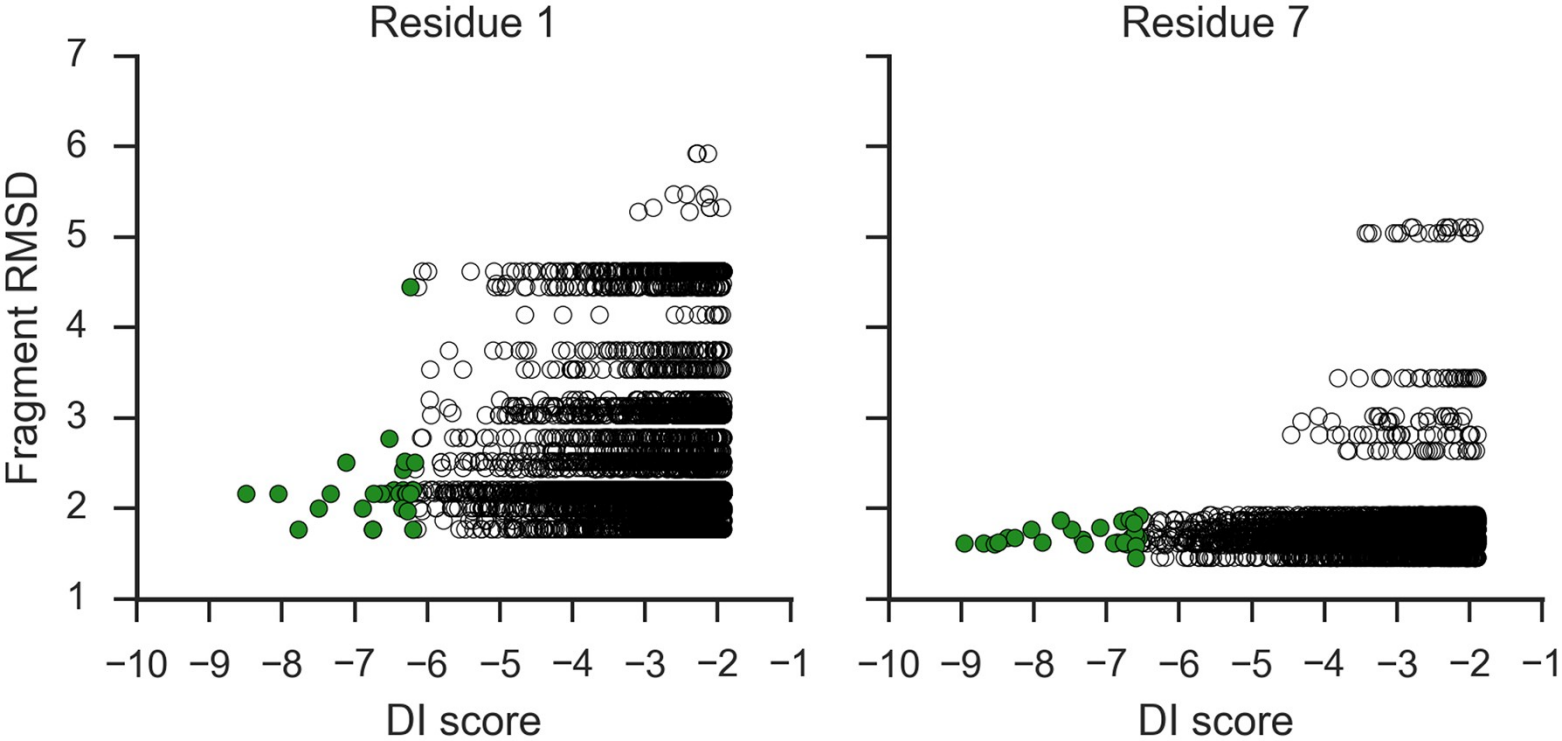
Correlation between the docking score (DI) and the RMSD of the fragments. Data for sequence windows 1 and 2 of 4i9o. Green: top 30 docked fragments by DI score.

Interestingly, as shown in Fig 2, evaluating docking fit with DI score often identified fragments of a low RMSD. To understand the general trend, for each sequence window we compared the fragment RMSD distributions of the 30 fragment structures from RFP and the top 30 docked fragments by DI score. Out of 144 windows from the 28 cases in the training set, for 83 (57.6%) windows the top 30 by DI score are either better (*p* <0.05 by the Mann-Whitney U test) or contained five or more fragments with an RMSD better than 3.5 Å (considered because there were cases where all 30 fragments from RFP were below 3.5 Å RMSD and no further improvement is possible by the DI score choice). This indicates that the DI score is detecting the increased binding affinity of the correct conformation when bound in the correct location, analogous to induced fit upon binding.

### Combining docked fragments

Docked fragments from each window were combined to form full-length IDP complexes, referred to as paths. First, we performed a pre-filtering of docked fragment pairs, which removes physically improbable pairs by considering mutual distances and angles; then, paths were assembled using an extend-and-cluster strategy (see Methods). This procedure effectively reduced the search space from as many as 10^41^ to the order of 10^5^ paths regardless of the length of the IDP (S2 Fig). Overall, the combination process successfully produced low RMSD paths. Out of the fourteen IDPs in the training set, for eleven bound and eight unbound receptors, paths with a 6.0 Å or lower RMSD were constructed (“Clustered paths” in S2 Table). Results were slightly worse for the testing set, an RMSD of below 6 Å was obtained for three bound and three unbound cases out of the eight IDPs.

### Path scoring and selection

For a complex, up to 1000 paths were chosen for further refinement. Paths were scored using a linear combination of four terms (Path Score): the energy score, representing the docking scores of fragments across all windows; the overlap score, evaluating how well the neighboring docked fragments fit into a continuous path; the cluster size, accounting for the consensus of docking poses; and the receptor score, which measures docking site consensus. Path Score selected more hits than any of the individual score components (S4 Table). On average, the minimum RMSD of selected paths was 6.7 Å for bound and 8.0 Å for unbound in the training set and 7.5 Å and 8.2 Å for bound and unbound in the testing set (S2 Table).

**Fig 3:**
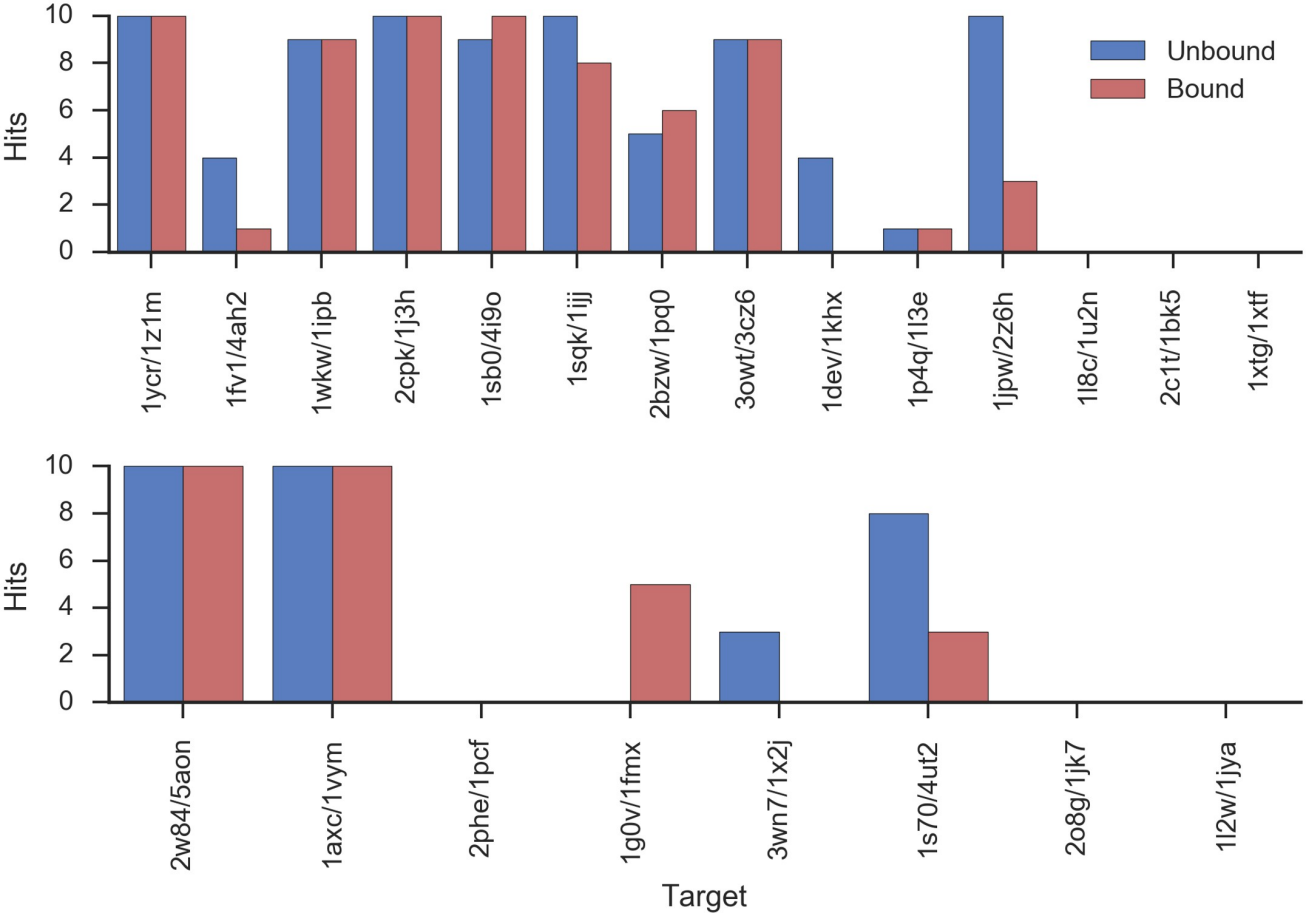
Selection of correct IDP conformation with Path Score. Hits: number of models with IDP RMSD < 6 Å in top 10 by Path Score. Blue: bound; red: unbound. Top: training complexes; bottom: testing complexes.

As in the situation in the docked fragment selection (Fig 2), it was observed that Path Score selected many models with IDPs of correct conformation (RMSD under 6.0 Å; Fig 3). Out of the fourteen pairs of targets in the training set, in ten/eleven cases for bound/unbound at least one of the top 10 models by Path Score has a correct IDP conformation. For the testing set, in four out of eight cases for both bound and unbound Path Score selected a correct IDP conformation within the top 10. These are again interesting results because Path Score mainly evaluates the binding affinity of a target IDP and its receptor, but also identifies IDPs of the correct conformation. Thus, in accordance with the biophysical mechanism, the binding affinity of the IDP is accumulated from the binding affinities of the individual segments and the conformation of IDPs is determined by binding.

### Refinement

**Fig 4:**
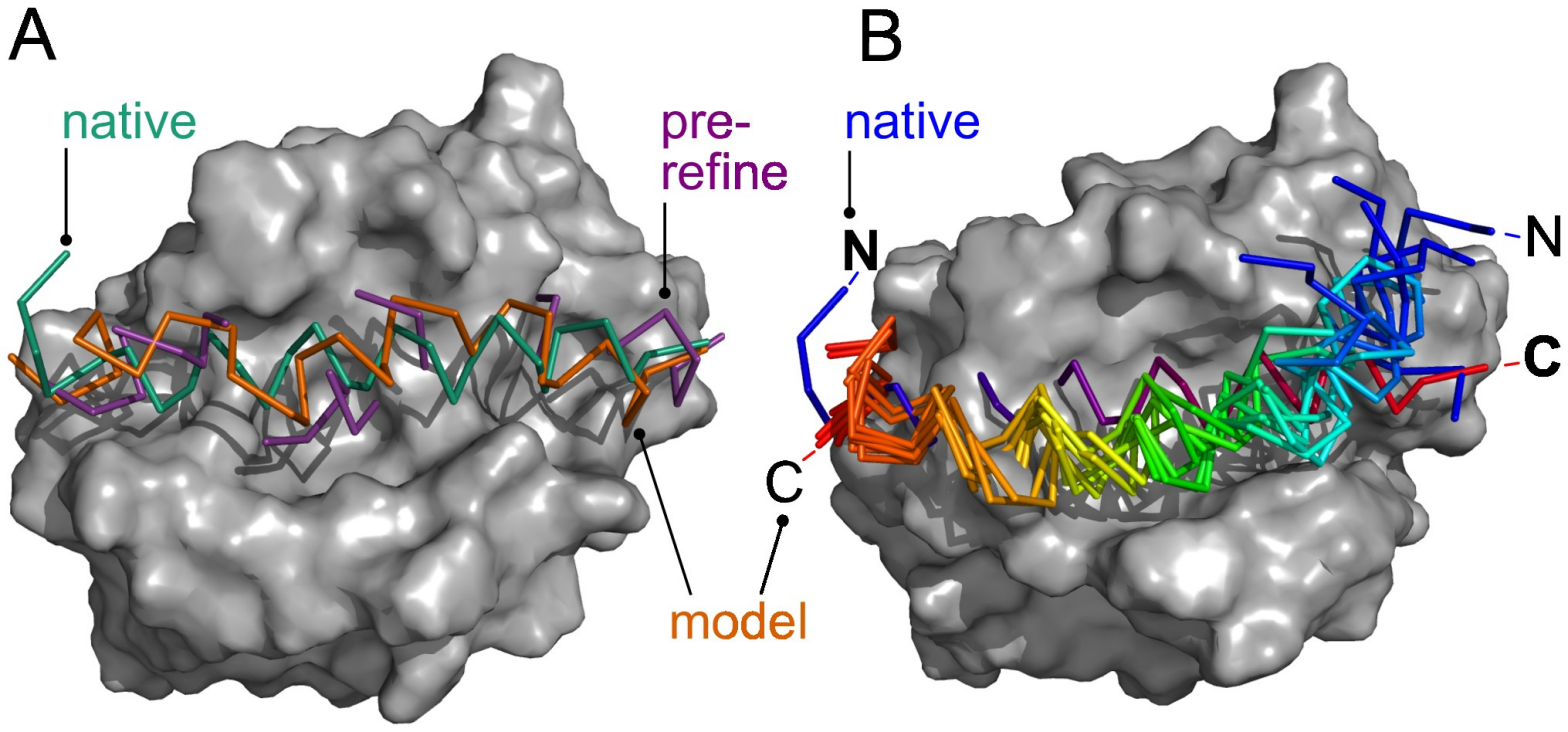
Complex between Bcl2-L-1 and BAD. **(A)**: A model of the bound structure (2bzw) before (purple) and after (orange) refinement vs. native (green). **(B)**: Unbound (1pq0); blue-to-red (N-terminus on the left): native BAD; rainbow: top 7 models of BAD.

Selected paths underwent structure refinement using constrained molecular dynamics, which connects neighboring fragments in a path and relaxes the overall IDP structure. An initial structure of a path was created by averaging the positions of the overlapping atoms (Fig 4A, purple). Multiple rounds of minimization were performed using tapering harmonic restraints to prevent excessive movement of fragments.

Refinement improved the protein-like nature of the combined fragments in a path. Before refinement, only 50.4 (48.2)% of ligand C_*α*_-C_*α*_ distances were between 3.75 and 4.0 Å in the training (testing) set, which was improved to 92.3 (95.8)% by the refinement (S3A Fig) with a small cost of deterioration of ligand RMSD (L-RMSD) for about half of the cases (S3B Fig). In parentheses, results for the testing set are shown. Refinement improved both L-RMSD and rank for some models, including the first hit for Bcl2-like protein 1 (Bcl2-L-1) and its antagonist (BAD; PDB ID 2bzw; Fig 4A). Originally, the path was ranked at 14 with a L-RMSD of 4.40 Å, which improved to rank 1 with L-RMSD 3.75 Å by the refinement.

### Model re-scoring and selection

Finally, refined models were re-ranked and selected using a composite score of DFIRE [51], ITScorePro [52], a molecular mechanics score [54], and GOAP [55] (Model Score). Model Score selected hits at a higher rank than the single scores (S5 Table).

**Fig 5:**
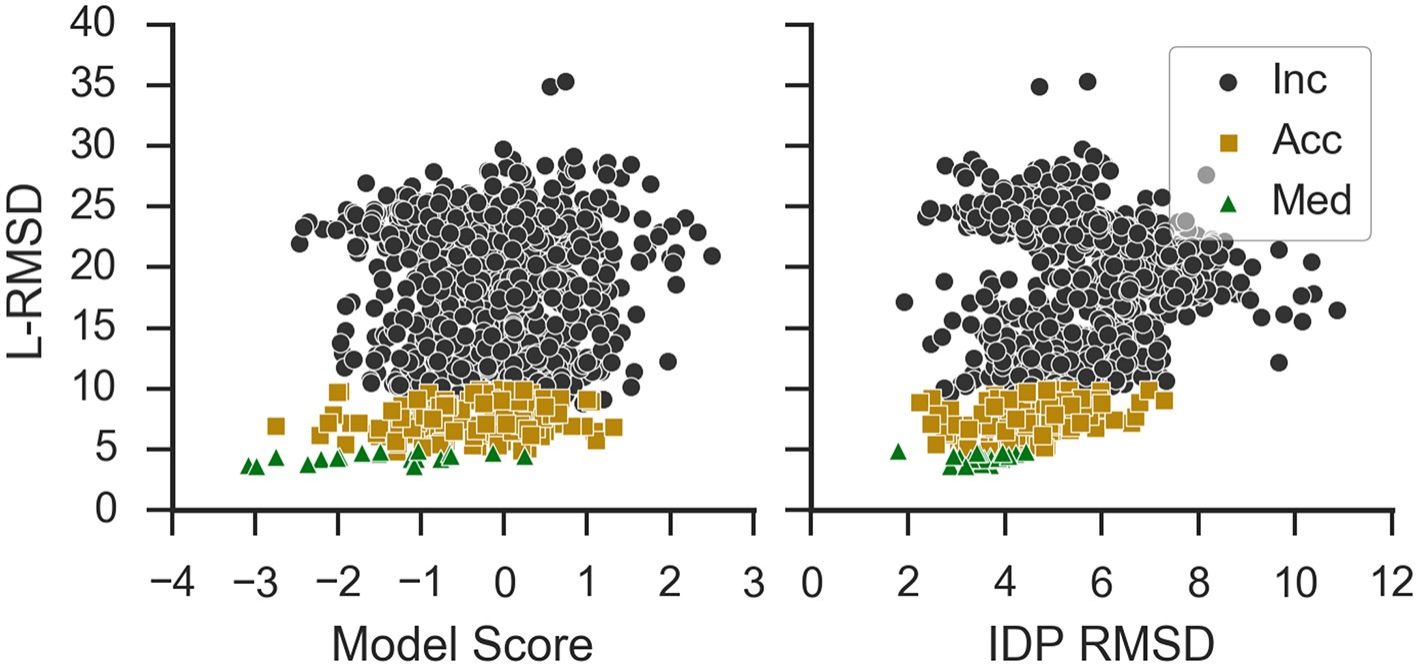
L-RMSD vs Model Score and IDP RMSD. Inc: incorrect; Acc: acceptable; Med: medium. PDB ID: 2bzw.

Model Score has moderate overall correlation to L-RMSD but often selected acceptable models with low scores (Fig 5, left panel) and successfully identified hits in many cases as we discuss in the next section. RMSD of IDPs only and L-RMSD of docked models only correlate for models with an L-RMSD less than 10 Å (Fig 5, right panel).

### Overall modeling performance

Tables 7 and 8 summarize prediction results on the training and testing sets, respectively, listing the rank of the first acceptable model (RFH) (the criteria for an acceptable model are shown in S1 Table) and *f*_*nat*_. On the training set (Table 7), IDP-LZerD produced at least one hit within the top 1000 models for thirteen bound and eleven unbound targets, and Model Score ranked hits within the top 10 for ten bound and five unbound cases. Notably, the rank 1 model was a hit for four complexes (three bound, one unbound). There was only one complex where no hits were produced for both bound and unbound (2c1t/1bk5). On the testing set (Table 8), IDP-LZerD produced at least one hit within 1000 models for almost all of the targets: all eight bound and seven unbound targets, and one top 1 hit for both bound and unbound. These fractions of top 1000 hits are higher than on the training set. Hits were ranked in the top 10 for two bound and three unbound cases. The fraction of top 10 hits (2/8, 25%, for bound cases) is lower than for the result observed on the training set (10/14, 71%), while higher for unbound cases (3/8, 37.5%) than the training set (5/14, 35.7%).

**Table 7:** Summary of modeling performance on training set.

Bound						Unbound				
PDB ID	L	RFH	RFH-B	*f*_*nat*_	BF10	PDB ID	RFH	RFH-B	*f*_*nat*_	BF10
1ycrA	15	1 (1)	1 (1)	0.42	1.00	1z1mA	6 (320)	6 (316)	0.13	0.85
1fv1AB	20	6	6	0.31	0.85	4ah2AB	1	1	0.40	0.90
1wkwA	20	16	15	0.39	0.45	1ipbA	53	53	0.24	0.60
2cpkE	20	4 (4)	3 (3)	0.56	1.00	1j3hA	15	15	0.17	0.35
1sb0A	25	3	3	0.32	1.00	4i9oA	136	134	0.18	0.40
1sqkA	25	14	14	0.36	0.24	1ijjA	9 (63)	9 (63)	0.55	0.92
2bzwA	27	1 (1)	1 (1)	0.49	1.00	1pq0A	-	-	-	0.22
3owtAB	27	6	5	0.33	0.90	3cz6AB	52	50	0.13	0.35
1devA	41	2	2	0.60	0.80	1khxA	16	16	0.22	0.59
1p4qB	44	5	5	0.27	0.82	1l3eB	3	3	0.25	0.86
1jpwA	45	1 (17)	1 (17)	0.38	0.92	2z6hA	2	2	0.23	0.83
1l8cA	51	33 (121)	32 (118)	0.26	0.57	1u2nA	16	16	0.32	0.71
2c1tA	51	-	-	-	0.06	1bk5A	-	-	-	0.06
1xtgA	59	5	3	0.17	0.61	1xtfA	-	-	-	0.24

RFH: rank of first acceptable (medium) hit; RFH-B: rank of first acceptable (medium) hit pre-filtered with BindML (S4 Fig); *f*_*nat*_: fraction of native contacts for the first acceptable hit. BF10: in top 10, highest fraction of ligand C_*α*_ atoms with L-RMSD ≤ 10 Å. Acceptable and medium defined in S1 Table.

**Table 8:** Summary of performance on test set.

Bound					Unbound			
PDB ID	L	RFH	*f*_*nat*_	BF10	PDB ID	RFH	*f*_*nat*_	BF10
2w84A	20	3 (35)	0.54	0.90	5aonA	6 (40)	0.19	0.30
1axcA	22	104	0.18	0.28	1vymA	81	0.17	0.39
2pheAB	26	11	0.25	0.23	1pcfAB	15	0.29	0.23
1g0vA	31	1 (1)	0.65	1.00	1fmxA	1 (4)	0.29	1.00
3wn7A	35	111	0.15	0.00	1x2jA	343	0.28	0.13
1s70A	39	252	0.31	0.33	4ut2A	-	-	0.08
2o8gA	40	17	0.32	0.60	1jk7A	37	0.19	0.35
1l2wAB	69	321	0.25	0.70	1jyaAB	2	0.21	0.74

RFH: rank of first acceptable (medium) hit; *f*_*nat*_: fraction of native contacts for the first acceptable hit. BF10: in top 10, highest fraction of ligand C_*α*_ atoms with L-RMSD ≤ 10 Å. Acceptable and medium defined in S1 Table.

Interestingly, for most of the cases in both training and testing set results, the acceptable models have a high *f*_*nat*_, much higher than the 0.1 minimum for an acceptable model defined by CAPRI (S1 Table). A high *f*_*nat*_ indicates that binding positions of IDPs are well reproduced in the models.

We also evaluated predictions in terms of the fraction of correctly placed ligand residues of the top 10 models (BF10). Unsurprisingly, the fraction is high for cases with hits ranked in the top 10. What is more interesting is that there are cases where targets that do not have any hits within the top 10 nevertheless have substantial BF10, which indicate largely correct models are ranked high. Such targets include 1wkw, 1l8c, 2o8g, and 1l2w from the bound targets and 1ipb, 4i9o, 1khx, and 1u2n from the unbound targets.

**Fig 6:**
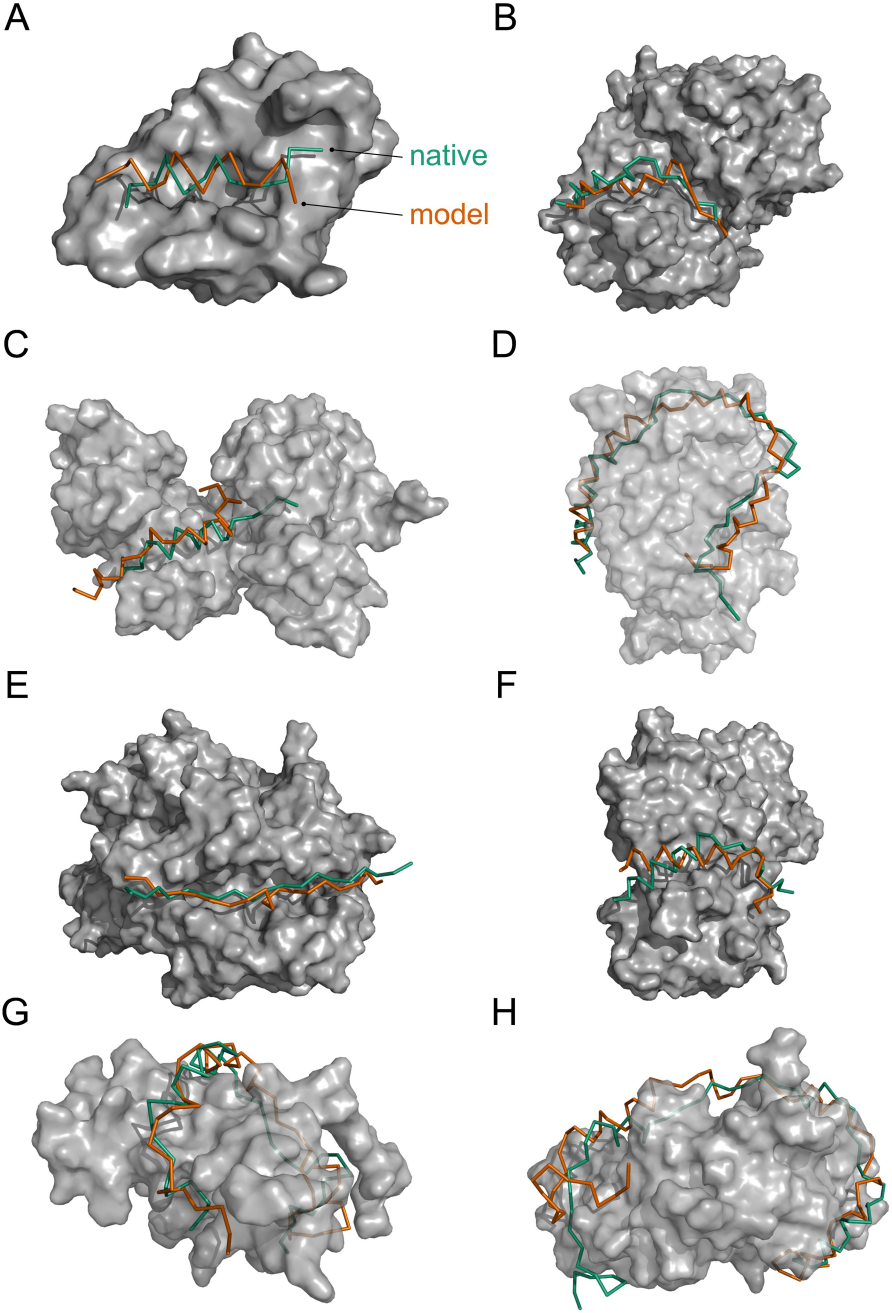
Examples of successful bound and unbound cases. Green: native IDP; orange: modeled IDP. a-d: bound cases; e-h: unbound cases. a: Rank 1 model of MDM2 with bound P53 (PDB ID: 1ycr). *f*_*nat*_ 0.42, I-RMSD 1.48 Å, L-RMSD 3.60 Å (medium quality). b: Rank 4 model of PKA C-*α* with bound protein kinase inhibitor *α* (2cpk). *f*_*nat*_ 0.56, I-RMSD 1.95 Å, L-RMSD 4.41 Å (medium quality). c: Rank 6 model of RAP1 with bound SIR3 (3owt). *f*_*nat*_ 0.33, I-RMSD 3.30 Å, L-RMSD 6.02 Å. d: Rank 5 model of BoNT/A with bound SNAP-25 (1xtg). *f*_*nat*_ 0.17, I-RMSD 3.79 Å, L-RMSD 9.22 Å. e: Rank 1 model of DRA/DRB5 with unbound myelin basic protein (4ah2). *f*_*nat*_ 0.39, I-RMSD 2.46 Å, L-RMSD 5.83 Å. f: Rank 9 model of *α*-actin-1 with unbound Cibulot (1ijj). *f*_*nat*_ 0.55, I-RMSD 2.51 Å, L-RMSD 5.15 Å. g: Rank 3 model of Cbp/p300 with unbound CITED2 (1l3e). *f*_*nat*_ 0.25, I-RMSD 6.31 Å, L-RMSD 7.43 Å. h: Rank 2 model of SycE with unbound YopE (1jya). *f*_*nat*_ 0.21, I-RMSD 5.44 Å, L-RMSD 9.97 Å.

Fig 6 shows examples of four bound and four unbound complexes with acceptable or better top 10 hits. The four bound cases shown, 1ycr, 2cpk, 3owt, and 1xtg, include two medium quality hits, with RMSD at the interface (I-RMSD) below 2.0 Å (1ycr and 2cpk), and the IDPs range in length from 15 to 59 amino acids. The four unbound cases, 4ah2, 1ijj, 1l3e, and 1jya, have IDPs between 20 and 69 amino acids. In all these examples, binding sites of the receptor proteins were accurately identified and overall docking structures were well predicted; often, even the pitch of the helices was reproduced. These examples demonstrate that IDP-LZerD can successfully select and combine docked fragments to produce accurate top 10 models for IDPs, even for cases with well over 30 amino acids.

### Using interface residue prediction

We also tested if binding residue predictions of receptor proteins is useful to improve model selection (Table 7). We used BindML [56], which predicts binding site residues from their mutation patterns. Models were first filtered by the agreement of binding residues to the BindML prediction (S4 Fig); then, the selected models were ranked by Model Score. Using BindML prediction (Table 7; RFH-B) did not make a large difference but slightly improved the model selection performance for 10 cases without worsening any cases.

### Influence of secondary structure prediction accuracy on final model quality

In this section we evaluated the impact of secondary structure prediction on the quality of final models in two ways. First, in S5 Fig we examined how the accuracy of the secondary structure of residues influenced the accuracy of the residue position (C_*α*_ RMSD) in the models. In the figure, for example, “HC” indicates cases where the native residue is helix and the modeled residue is coil. It turned out that correctly predicted helix residues (class “HH”) have lower mean C_*α*_ RMSD, e.g. are more accurate, than other classes (one-way ANOVA *p* = 1 × 10^−35^ and Tukey’s range test).

Next, in S6 Fig, we addressed the influence of the secondary structure prediction agreement on the C_*α*_ RMSD of residues. The X-axis shows the number of secondary structure prediction methods that agree (e.g. consensus) on the correct secondary structure of residues and the Y-axis is the C_*α*_ RMSD of residues in the models. Residues where none of the four secondary structure prediction methods predict the correct secondary structure (consensus 0) have higher (worse) mean C_*α*_ RMSD than other residues (one-way ANOVA *p* = 1 × 10^−11^ and Tukey’s range test). Thus, we see some influence of the accuracy of predicted secondary structure to the quality of the final model with statistical significance, but as seen from the figures, difference was not very large. In IDP-LZerD, the fragment generation procedure creates fragments of all three secondary structure classes even if none of the methods predict the correct class to minimize the impact of incorrect secondary structure prediction.

### Comparison with existing methods

To further examine performance of IDP-LZerD, we compared modeling results with other methods. While no other methods are designed to model complexes involving long IDPs, some peptide-protein modeling software can use relatively long peptides. We compared IDP-LZerD with CABS-dock [27] and pepATTRACT [26], because as seen in Table 1, these two do not require the binding site as input and the programs are available for us to run. The CABS-dock web server outputs 10 docking models for a peptide up to 30 amino acids while the pepATTRACT web server outputs 50 docking models and does not explicitly limit the length of the peptide. The performance was compared on the eleven bound and unbound complexes with IDPs up to 30 amino acids in Tables 2 and 3.

Within the top 10, CABS-dock had hits for six bound cases and four unbound cases, pepATTRACT had hits for three bound cases and one unbound case, and IDP-LZerD had hits for seven bound and four unbound cases (Table 9). The longest IDP successfully modeled by CABS-dock was 26 amino acids and the longest IDP successfully modeled by pepATTRACT was 22 amino acids. In contrast, IDP-LZerD had top 10 hits for the longest IDPs in this table (27 amino acids; Table 9) in addition to even longer IDPs in the full dataset (Tables 7 and 8). Therefore, overall IDP-LZerD showed better performance than the two methods compared.

**Table 9:** Performance comparison of IDP-LZerD to CABS-dock and pepATTRACT on ≤ 30 amino acid IDPs.

Bound		Top 10 hits			Unbound	Top 10 hits		
PDB ID	L	CABS-dock	pepATTRACT	IDP-LZerD	PDB ID	CABS-dock	pepATTRACT	IDP-LZerD
1ycrA	15	4	7/4**	5/4**	1z1mA	4	-	2
1fv1AB	20	2	1	1	4ah2AB	1	2	7
1wkwA	20	-	-	-	1ipbA	-	-	-
2cpkE	20	2	-	1/1**	1j3hA	-	-	-
2w84A	20	3/1**	-	2	5aonA	6/1**	-	1
1axcA	22	-	1	-	1vymA	-	-	-
1sb0A	25	1	-	3	4i9oA	-	-	-
1sqkA	25	-	-	-	1ijjA	-	-	1
2pheAB	26	1	-	-	1pcfAB	2	-	-
2bzwA	27	-	*n/a*	7/5**	1pq0A	-	*n/a*	-
3owtAB	27	-	-	1	3cz6AB	-	-	-
Total hits		6/1**	3/1**	7/3**		4/1**	1	4

Table only includes complexes with IDPs up to 30 amino acids because the CABS-dock web server has a maximum length of 30 residues. *n/a* indicates that pepATTRACT did not run due to missing receptor residues.—indicates no hits in the top 10.

** indicates medium-quality hits. For example, 5/4** indicates that out of the top 10 models, 5 acceptable models were produced, among which 4 of them had medium quality. The CABS-dock web server outputs 10 models and pepATTRACT outputs 50 models (results are shown for the first 10).

In addition, we compared the performance of IDP-LZerD to the previously published results of MD-based peptide-protein modeling methods [17–19]. The protein-peptide complexes used in their literature range from 2–15 amino acids. Among their datasets, we ran IDP-LZerD on all cases with 11 or more amino acids and unbound receptors, for a total of eight cases (S6 Table). IDP-LZerD produced acceptable models in the top 10 for five out of eight cases with a sixth case having an acceptable model at rank 306 (Table 10). For the two cases with no hits, 2am9 and 1b9k, paths with 5 Å RMSD were created in Step 3 (Fig 1) but not selected for refinement. IDP-LZerD and AnchorDock produced the same number of hits, but the models produced by AnchorDock have a lower RMSD. The results indicate an advantage of MD over coarse-grained approach for short peptides. They also suggest a potential improvement of IDP-LZerD by employing MD for the initial fragment-docking step, although it would take significantly more computational time than the current procedure.

**Table 10:** Performance of IDP-LZerD on ≥ 11 amino acid protein-peptide complexes from MD test sets.

Unbound		Anchordock^a^			Dagliyan^b^	IDP-LZerD		
PDB ID	L	Rank	RMSD (Å)	*f*_*nat*_	RMSD (Å)	Rank	RMSD (Å)	*f*_*nat*_
2am9	15	14	2.2	0.81	*n/a*	-	-	-
1jbe	15	3	1.5	0.82	10.5	1 (83)	8.9 (6.2)	0.23 (0.64)
2j2i	14	-	-	-	*n/a*	9 (42)	8.2 (4.7)	0.13 (0.31)
1oot	12	3	1.7	0.77	*n/a*	1 (295)	7.5 (3.6)	0.30 (0.70)
2aa2	12	1	2.0	0.81	*n/a*	306	5.0	0.28
1i7g	12	4	2.2	0.73	*n/a*	1 (11)	6.0 (4.39)	0.28 (0.39)
1b9k	12	-	-	-	*n/a*	-	-	-
1rwz	11	6	1.3	0.74	5.77	3	6.9	0.26

^a^: Values from Table 2 in [19];

^b^: values from Table 1 in [18].

For IDP-LZerD, results are shown for the first acceptable (medium) hit. Dash (-) indicates no hits; *n/a* indicates that the complex was not part of the dataset. All RMSD values are for ligand backbone atoms.

### Case studies

**Fig 7:**
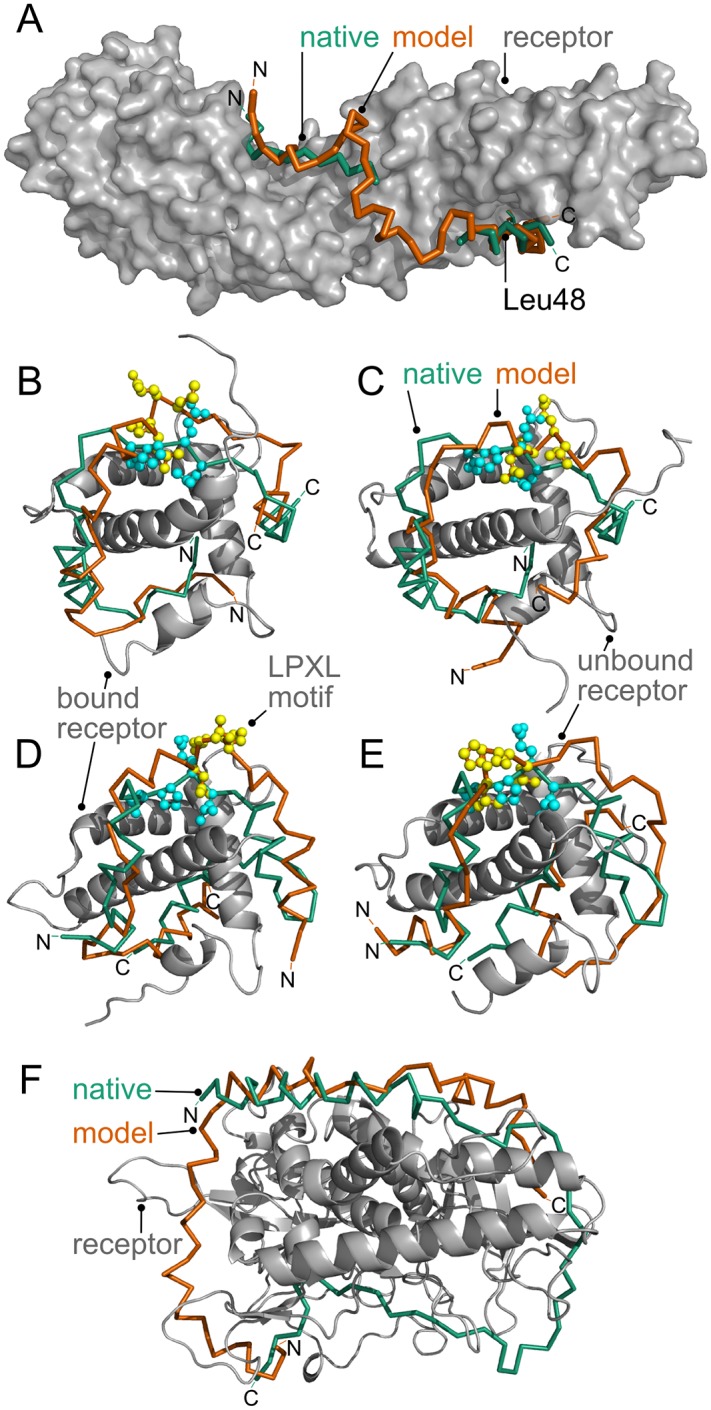
Biological case studies. **A**: *β*-catenin in complex with TCF7L2. Green: native TCF7L2; orange: rank 1 model of TCF7L2; *f*_*nat*_ 0.38, I-RMSD 2.85 Å, L-RMSD 7.94 Å. PDB ID: 1jpw. **B-E**: Human and mouse Cbp/p300 TAZ1 domain in complex with CITED2 and Hif-1*α*. Green/cyan: native CITED2/Hif-1*α*; orange/yellow: model CITED2/Hif-1*α*. Ball and stick: LPXL motif. **B-C**: Human TAZ1 and CITED2. **B**: bound (1p4qB); rank 5 model; *f*_*nat*_ 0.27, I-RMSD: 4.2 Å, L-RMSD: 7.6 Å. **C**: unbound (1l3eB); rank 9 model; *f*_*nat*_ 0.17, I-RMSD: 7.1 Å, L-RMSD: 9.6 Å. **D-E**: Mouse TAZ1 and Hif-1*α*. **D**: bound (1l8cA); rank 16 model; *f*_*nat*_ 0.05, I-RMSD 11.7 Å, L-RMSD 20.1 Å. **E**: unbound (1u2nA); rank 9 model; *f*_*nat*_ 0.20, I-RMSD 6.4 Å, L-RMSD 10.4 Å. **F**: Unbound complex between BoNT/A and sn2. Green: native sn2; orange: rank 1 model of sn2; *f*_*nat*_ 0.00, I-RMSD 15.7 Å, L-RMSD 38.2 Å. Receptor PDB ID 1xtfA (unbound).

In addition to the other successful cases, we chose four cases to discuss, which illustrate the usefulness of IDP-LZerD models. In some disordered PPIs, the IDP forms secondary structure in the bound form that is not seen in isolation. The interaction between *β*-catenin and Transcription factor 7-like 2 (TCF7L2), which is involved in the Wnt signal transduction pathway, is such an example. In isolation, TCF7L2 exhibits circular dichroism spectra consistent with 96% random coil and 4% *β*-sheet, indicating that it is intrinsically disordered [57]. In contrast, the crystal structure of the complex (1jpw) shows a C-terminal helix (residues 40–50), which was correctly predicted by the secondary structure methods and many models by IDP-LZerD. For both bound (1jpw) and unbound (2z6h) receptors, the overall complex was well-modeled (RMSD at the interface, I-RMSD: 2.85 Å for bound and 4.50 Å for unbound) with the structure and location of the C-terminal helix and hotspot residue Leu48 (full atom L-RMSD 1.43 Å) predicted very well in the bound case (Fig 7A). Interestingly, among 1000 docking models generated, Leu48 was the most frequent contact in both the bound and unbound cases, appearing in 956 models for bound and 944 models for unbound, compared to an average of 685 and 696, respectively (S7 Fig). There are two more experimentally verified hotspot residues in the IDP, Glu17 and Asp16 [57]. Glu17 was in contact with the receptor in both bound and unbound cases in more than the average number of models, but Asp16 did not stand out (S7 Fig).

The next examples are complexes between CREB-binding protein (Cbp)/p300 TAZ1 domain and its disordered regulator proteins, hypoxia inducible factor 1-*α* (HIF-1*α*) and its competitive inhibitor, Cbp/p300-interacting transactivator 2 (CITED2). HIF-1*α* and CITED2 are different lengths, have only 12.5% sequence identity, and bind differently to the Cbp/p300 TAZ1 domain (in Fig 7**B**, the N-terminus of CITED2 is at the bottom right while in panel **D** the N-terminus of HIF-1*α* is at the bottom left. The TAZ1 domain is shown in the same orientation in all panels). Nevertheless, the IDPs share a conserved binding motif (LPEL in CITED2, LPQL in Hif-1*α*, referred as LPXL) [58]. We docked two complexes: CITED2 with human TAZ1 (bound, 1p4q) and HIF-1*α* with mouse TAZ1 (bound, 1l8c; unbound, 1u2nA). Because the human TAZ1 domain does not have an available unbound structure, we used its structure in complex with HIF-1*α* (1l3eB) for the unbound case, which has a binding site RMSD of 5.11 Å to the bound form with CITED2. Remarkably, the prediction was accurate not only for the bound (Fig 7B), but also for the unbound case (Fig 7C). Both leucines in the LPXL motif, Leu243 and Leu246, were experimentally verified as hotspot residues by mutagenesis [59], but differ in contact consensus among the 1000 models. Leu243 has above-average counts (rank 11, 814 models, average 679 for bound and rank 8, 851 models, average 713 for unbound) while Leu246 has below-average counts (rank 36, 571 models for bound and rank 40, 486 models for unbound; S8 Fig). For the mouse homolog, the bound case had no model under 12.6 Å L-RMSD in the top 10. The rank 16 model shown had L-RMSD 20.1 Å, but the LPXL motif is located roughly at the correct position (Fig 7D). The unbound case had no model with L-RMSD under 10.4 Å in the top 10. However, HIF-1*α* was bound to almost the right location in the rank 9 model (Fig 7E), where the fraction of correctly placed ligand residues was 0.71 and the L-RMSD of the LPXL motif was 3.7 Å. In addition, the residue Leu795, which was experimentally determined to be a hotspot residue [60], has high contact consensus for both bound and unbound (rank 5, 911 models, average 734 for bound and rank 8, 881 models, average 694 for unbound; S9 Fig) in the final 1000 models. Thus, in these four models the IDPs were bound almost at the correct place with the LXPL motif predicted particularly well.

Finally, we discuss two cases where predictions did not yield acceptable quality models. The first case is the complex between Bcl2-like protein 1 (Bcl2-L-1) and Bcl2-associated Antagonist of cell Death (BAD). While the bound receptor had an excellent result with a medium quality model at rank 1 (2bzw; Table 7, Fig 4a), the unbound receptor (1pq0) had no hits. However, visual inspection of the top-ranked models shows that the rank 1 to 7 models have a correct IDP conformation and binding site; however, the IDP is rotated by 180° within the binding site (Fig 4b). Thus, the scoring functions detected a region of affinity but lacked the specificity to distinguish the correct orientation.

The last example is a complex between botulinum neurotoxin type A (BoNT/A) and the N-terminal SNARE domain of SNAP25 (sn2). BoNT/A causes paralysis by cleaving SNARE proteins which impairs neuronal exocytosis [61]. Using the bound receptor (PDB ID: 1xtgA), the structure was correctly predicted at rank 5 (Fig 6d). However, with the unbound receptor (1xtfA), no hits were found. In the rank 1 model of the unbound case, while the IDP shows a substantial registration shift, the model occupies 32.6% of the binding groove (top in Fig 7F; measured by the number of receptor residues within 5 Å of both IDPs). Thus, even in cases where no hits are produced, the produced models are reasonable and capture characteristic binding modes of IDPs on their receptors.

## Discussion

The current study presents for the first time that PPIs with long IDPs can be modeled with reasonable accuracy. By taking advantage of the crucial observation that disordered proteins tend to bind in continuous segments, the procedure is not only more computationally feasible but also functions similarly to the biophysical mechanism of IDP association. The prediction by IDP-LZerD was successful for the majority of the complexes tested, including unbound cases. The study further observed that the correct conformation of IDPs are often identified by evaluating docking scores with receptor proteins.

A major challenge in modeling IDP interactions is the existence of fuzziness, where the IDP continues to exhibit multiple conformations in the bound state [11]. Two cases in the dataset we used are listed as fuzzy complexes in the FuzDB [11]: 1g0v (FuzDB ID FC0018) and 3wn7 (FuzDB ID FC0076). IDP-LZerD managed to obtain a rank 1 medium hit for 1g0v (Table 8), while for 3wn7 IDP-LZerD produced an acceptable model at a low rank. It is particularly challenging to predict complexes where an IDP binds with two or more regions separated by loop regions that do not have direct contact its receptor (clamp complexes [10]), because IDP-LZerD is based on the assumption that each segment of the IDP is in contact with the receptor.

There are several other potential areas of improvement for the method. Docking larger fragments in cases where the structure of the fragments can be predicted with confidence could improve accuracy. It is also interesting to employ a coarse-grained model such as CABS [62] for generating fragment conformations and for more efficient structure refinement. In addition, explicit consideration of receptor flexibility could improve performance, although the soft surface representation used by LZerD already accounts for some degree of receptor flexibility. A key feature would be the ability to handle phosphorylated residues, as IDPs are frequently sites of post-translational modification and some complexes. This would require consideration of the effect of phosphorylation on secondary structure in addition to modification of the docking and scoring protocols. Another potential area of improvement is to guide docking by considering known or predicted hotspot residues on both IDPs and receptor proteins. Methods that could detect hotspots include computational alanine scanning [63] or applying a statistical scoring function [51, 52] on a per-residue basis. Alternatively, as we showed in the case studies (S7, S8 and S9 Figs) some promise was shown that hotspot residues could be predicted by taking consensus binding sites from ensembles of docking models. Accurate detection of hotspot residues could also lead to improved performance for fuzzy complexes, particularly the clamp class where two or more stably bound regions of the IDP are separated by fuzzy regions.

Disordered PPIs are involved in important roles in various pathways and diseases. Overall, the work opens up a new possibility of modeling disordered protein interactions, providing structural insights for understanding the molecular mechanisms and malfunctions of these interactions, which are difficult to obtain by both experimental means and conventional computational protein docking methods.

## Methods

### Selection of datasets of protein complexes

Protein complexes containing IDPs with diverse functions and lengths were selected for developing and testing IDP-LZerD. Candidate complexes were found from reviews of disordered protein complexes [2, 6]. In addition, cases were found in databases of eukaryotic linear motifs (ELMs) [53] and fuzzy complexes (FuzDB) [11]. For each case, disorder was verified by searching the literature for experimental evidence and DisProt [13] for a corresponding entry (if available). Each PDB file was visually inspected and the case was removed if the residues annotated as disordered were missing or phosphorylated. The remaining proteins were divided into a training set of 14 complexes (Table 2) and a test set of 8 complexes (Table 3). For bound complexes, unbound structures of the receptor, which were solved without the IDP, were found by searching PDB entries of the same UniProt ID as the receptor protein(s). Docking using an unbound structure of the receptor protein would be more similar to a realistic scenario where the bound structure is unknown. If no PDB entries had the same UniProt ID, PDB entries with 90–100% sequence identity were used. Gaps of up to 16 amino acids were rebuilt using MODELLER [43].

In addition to the bound and unbound dataset described above, an additional dataset of 9-residue intrinsically disordered region (IDR) fragments was constructed from the database of eukaryotic linear motifs (ELM) [53]. To select disordered fragments, 442 proteins with structures in the PDB were cross-referenced against DisProt [13] using the Uniprot [64] ID, yielding 26 candidate complexes. By manual inspection of the PDB files, cases were removed if they were redundant (using PISCES [65] with a 25% sequence identity cutoff) with the full-length training set (Table 2; 5 cases), other proteins within the set (6 cases), phosphorylated (4 cases), only had one chain (2 cases), or had fewer than 9 residues around the ELM resolved (4 cases). In addition, cases were added using other chains (1 case) or adjacent to the ELM and also annotated as disordered (5 cases). The 11 cases of 9-residue fragments are listed in Table 5.

### IDP fragment structure prediction

For each IDP sequence, we provided four independent secondary structure predictions, from PSIPRED [47], JPRED [44], Porter [45], and SSPro [46], each of which was used separately to generate one fourth of the fragments output by Rosetta Fragment Picker (RFP). RFP was configured to output 30 fragments for a window (S1 Fig). RFP produces fragments of each secondary structure class in proportion to its confidence score. For predictions by Porter and SSPro, which do not output confidence scores, we used 0.67 for the predicted secondary structure class and 0.15 for the other classes. Thus, fragments of all three secondary structure classes are obtained even in cases where the secondary structure prediction has strong consensus for one class. From the C_*α*_ coordinates of a fragment produced by RFP, the full atom backbone and side-chains were constructed using Pulchra [66] and OSCAR-star [67, 68], respectively.

### Docking fragments to receptor

LZerD is a shape-based, rigid body docking algorithm [35]. For two input protein structures, LZerD generates many docking poses by geometric hashing and evaluates docking models using a scoring function that considers surface shape matching. Surface shape complementarity is evaluated using a mathematical surface descriptor, 3D Zernike Descriptor (3DZD) [69, 70]. Since 3DZD controls the level of surface smoothness, some degree of protein flexibility is considered in LZerD. 50,000 docking models were generated by LZerD for each fragment structure. Docked fragments were clustered with an RMSD cutoff of 4.0 Å and cluster centers were chosen using the LZerD score. The cluster centers were scored with ITScorePro [52] and the top 1,000 scoring fragments were pooled for each of 30 fragments of a window. Out of the 30,000 (1,000*30) docked fragments for each window, 4,500 docked fragments with the lowest DI score (the sum of the Z-scores of ITScorePro and DFIRE [51]) were kept (S3 Table).

### Combining docked fragments

**Fig 8:**
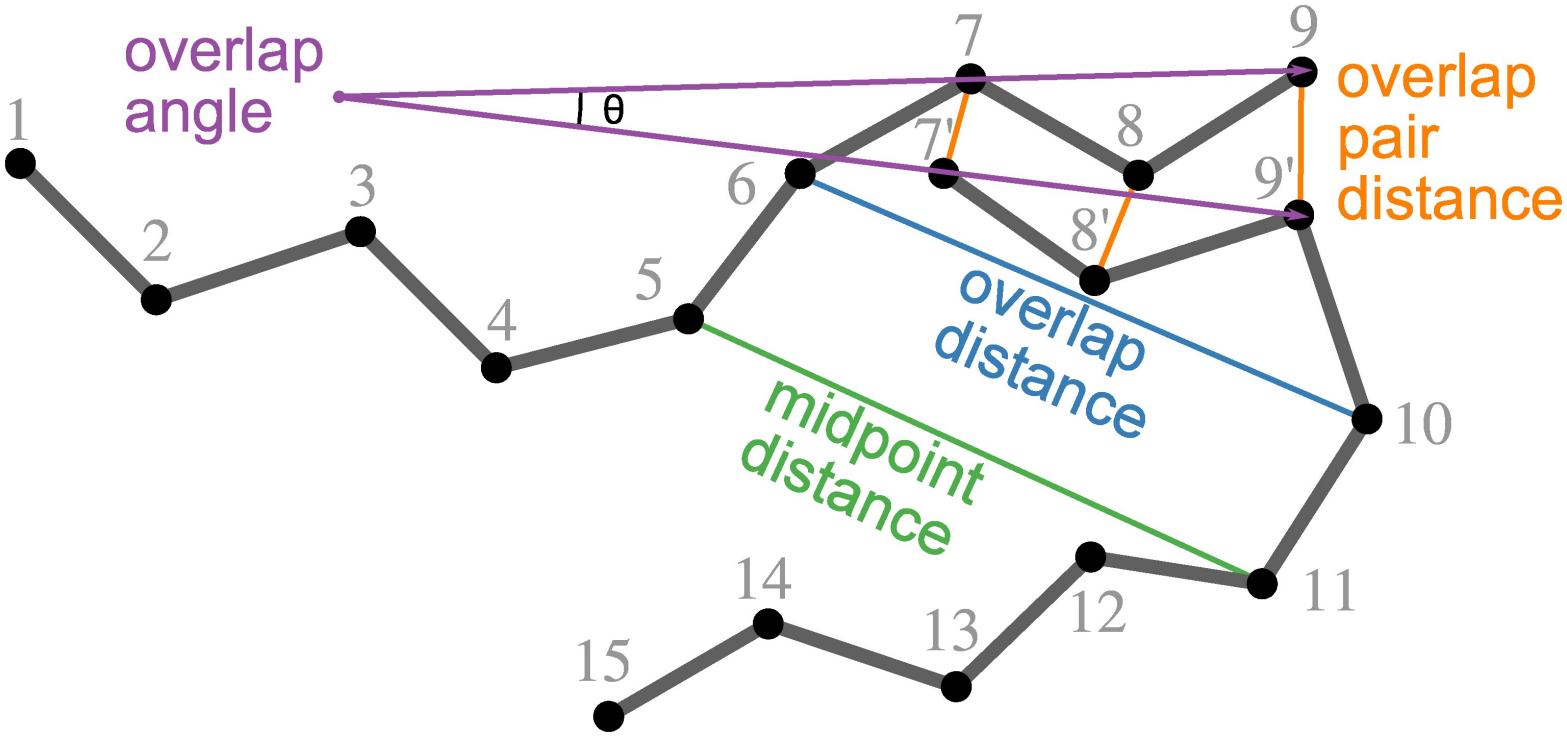
Fragment geometry subject to cutoffs. Midpoint distance: between the C_*α*_ atoms of the middle residues of two fragments; overlap distance: between the C_*α*_ atoms of the residues before and after the overlapping residues; overlap pair distance: between the corresponding N, C_*α*_, C_*β*_, and C atoms of the three overlapping residues; overlap angle: formed by the vectors from the N atom of the first overlapping residue to the C atom of the third overlapping residue.

By choosing one docked fragment from each window, conformations of the full length IDP, referred to as paths, were created. Prior to the path search, distance and angle cutoffs (Fig 8) were applied to remove physically improbable pairs of docked fragments. Distance cutoffs were determined heuristically from the observed distributions in IDPs in DisProt [13] (S10 Fig). Docked fragment pairs from all pairs of windows were removed from consideration if they are too close, i.e. an atom distance less than 3 Å or fragment midpoint distance less than 6.5 Å for neighboring windows and 3.8 Å otherwise. Pairs were also removed if their midpoint residues are too distant, more than 18.5 Å times the separation between the windows (e.g. 2 for windows A and C). Also, to ensure that fragments from neighboring windows can be connected in the refinement stage, pairs are removed if they do not satisfy the following criteria: the overlap residue distance (min. 5.2 Å, max. 13.6 Å), the overlap atom pair distances (max. 6 Å for all atoms or 10 Å for any atom), and the overlap angle (cos *θ* ≥ 0.1 so that only smoothly connected turns are included).

Paths were assembled by first combining allowed pairs of docked fragments from the first two sequence windows and clustering them with a cutoff of 4.0 Å. Paths were extended to three windows, clustered again, and this process was repeated until all windows were added.

### Path scoring

A path was evaluated by Path Score, a linear combination of the Z-scores of four component scores: energy score (*S*_*E*_), overlap score (*S*_*O*_), cluster size (*S*_*C*_), and receptor score (*S*_*R*_):

$$\begin{array}{cc} S_{path}= & w_{5}\left(w_{1}Z\left(S_{E}\right)+w_{2}Z\left(S_{O}\right)+w_{3}Z\left(-S_{C}\right)+w_{4}Z\left(-S_{R}\right)\right)\\ & +\left(1-w_{5}\right)\min\left\{Z\left(S_{E}\right),\;Z\left(S_{O}\right),\;Z\left(-S_{C}\right),\;Z\left(-S_{R}\right)\right\} \end{array}$$ (1)

where Z represents the Z-score across all paths in one complex. The lowest Z-score among the scores was included as an additional term because in some cases a good model is only detected by some of the scores. *S*_*C*_ and *S*_*R*_ are inverted so that a low Path Score is a favorable score. *S*_*E*_ is the average of the binding scores (DI score) of docked fragments in the path. *S*_*O*_ is the average of mean square distance (MSD) between the overlapping residues between consecutive fragments:

$$S_{O}=\frac{\sum_{n=1}^{|W|-1}\;MSD\left(n\right)}{|W|-1},where$$ (2)

$$MSD\left(n\right)=\sum_{r=1}^{v}\;\sum_{a\in A}\;\frac{\left|\right|X_{n,r+l-v,a}-X_{n+1,r,a}{\left|\right|}^{2}}{v\times |A|}$$ (3)

and *W* is the set of windows, *A* is a set of N, C_*α*_, C_*β*_, and C atoms of an overlapping residue, *v* is the overlap size (=3), *l* is the window length (=9), and X_*n*,*r*,*a*_ is the 3D coordinates of atom *a* of residue *r* of the docked fragment for window *n*. The *C*_*β*_ atom is included in the computation of *S*_*E*_ to account for rotational as well as translational congruency. For glycine, a virtual *C*_*β*_ was constructed. *S*_*C*_ is defined as the number of members in the path’s cluster. *S*_*R*_ for a path considers whether the IDP binds to surface regions of the receptor that are also bound by other paths. For a surface residue of the receptor, the number of paths that bind to the residue (minimum heavy atom distance ≤ 5.0 Å) was counted (called the number of occupying paths, *N*_*op*_), and *S*_*R*_ of a path is defined as the sum of *N*_*op*_ of the binding residues of the path.

Weights were trained using a grid search from 0.1 to 1.0 with an increment of 0.1 and $\sum_{1}^{4}\;w=1$. The weight for the lowest Z-score, *w*_5_, was trained in a second grid search. Weights were chosen that maximized the minimum recall of the targets used (S4 Table). Recall is the number of hits retrieved by a given score divided by the total number of hits. Hits were defined as paths having pooled RMSD ≤ 10 Å. The pooled RMSD for a given path is defined as $\sqrt{\sum_{n\in W}\;d_{n}^{2}/\left|W\right|}$ where *W* is the set of windows and *d*_*n*_ is the backbone L-RMSD of the docked fragment for window *n*, computed using only residues present in the crystal structure. The final weights for *w*_1_ through *w*_5_ were 0.5, 0.1, 0.3, 0.1, and 0.3 (Eq 1), with minimum recall of 2.8%. In addition, to validate the trained weights, we further performed a 2-fold cross validation by splitting the training dataset (Table 2) into two groups. The results are shown in S7 Table. The weights obtained by training on group 1 were 0.4, 0.3, 0.2, 0.1, and 0.1. The minimum recall observed on the group 2 set when predicted by using these weights was was 2.4% (obtained for 1j3hA). The weights obtained on group 2 were 0.3, 0.2, 0.4, 0.1, and 0.1, and the minimum recall when applied to the group 1 targets was 1.1%, observed for both 1devA and 1khxA. Thus, the final weights used in this study and the minimum recall were not largely different from what was observed in the 2-fold cross validation. For each complex, the 1000 paths with the lowest Path Score were kept for refinement, described in the next section.

### Refinement

The selected paths were refined using molecular dynamics simulation. FACTS implicit solvation [71] was used. For minimization, all atoms of the receptor were fixed. With the ligand under a harmonic constraint of 50 kcal/mol/Å^2^, the complex was minimized using 100 steps of the steepest descent (SD) algorithm followed by 100 steps of the adopted basis Newton-Raphson algorithm (ABNR). This was followed by four rounds of 100 steps of ABNR minimization with ligand constraints of 40, 30, 20, and 10 kcal/mol/Å^2^. Next, the constraints were only placed on the backbone atoms of the ligand. Three rounds of 100 steps of ABNR minimization were run with ligand backbone constraints of 10, 5, and 1 kcal/mol/Å^2^. The final minimization round was 5000 steps of ABNR minimization with no ligand constraints. Finally, the structure was equilibrated for 40 ps using a 2 fs timestep, fixed hydrogen covalent bond lengths, and a harmonic constraint of 10 kcal/mol/Å^2^ on all C_*α*_ atoms. The molecular dynamics simulation protocol was performed using CHARMM [72] but will also run using the academic free version charmm and could be implemented using other standard molecular dynamics software that implements harmonic constraints.

### Model re-scoring

Refined models were re-ranked using Model Score, an integrated score of ITScorePro [52], DFIRE [51], a molecular mechanics score [54], and GOAP [55]:

$$\begin{array}{cc} S_{model}= & w_{5}\left(w_{1}Z\left(\text{ITScorePro}\right)+w_{2}Z\left(\text{DFIRE}\right)+w_{3}Z\left(\text{MolMech}\right)+w_{4}Z\left(\text{GOAP}\right)\right)\\ & +\left(1-w_{5}\right)\min\left\{Z\left(\text{ITScorePro}\right),\;Z\left(\text{DFIRE}\right),\;Z\left(\text{MolMech}\right),\;Z\left(\text{GOAP}\right)\right\} \end{array}$$ (4)

The lowest Z-score among the scores was included as an additional term because in some cases a good model is only detected by some of the scores. Weights were trained using a grid search with increments of 0.1 and $\sum_{1}^{4}\;w=1$. The weight for the lowest Z-score, *w*_5_, was trained in a second grid search. Weights were chosen that minimized the mean rank of first hit (RFH) across all complexes used (S5 Table). Hits were determined following the CAPRI criteria [39]. RFH is the numerical rank of the first model with CAPRI classification of acceptable or higher quality (S1 Table). The final weights for *w*_1_ through *w*_5_ were 0.1, 0.2, 0.3, 0.4, and 0.3 (Eq 4), with mean RFH of 11.3. To further confirm the validity of the trained weights, we performed an additional 2-fold cross validation (S7 Table). The weights obtained on the group 1 set were 0.2, 0.4, 0.1, 0.3, and 0.3 and the mean RFH observed on the group 2 set when the predictions were made using the group-1 weights was 16.5. The second group weights were 0.4, 0.1, 0.1, 0.4, and 0.1 and the mean RFH observed on the group 1 set by using the second group weights was 16.4. RFH values obtained from this 2-fold cross validation (S7 Table) were very similar to the values reported in Table 7, which indicates that the final weights were reasonably trained and capture the score landscape of the docking models well: out of 28 targets, RFH results were either the same or within a difference of 5 ranks for 22 targets.

### Computational time and availability

Docking one fragment to a receptor structure takes 2–4 hours on a single CPU. Thus, the docking step (step 2 in Fig 1) takes about 120 CPU hours for a small receptor with a short IDP and as many as 1000 CPU hours for a large receptor with a long IDP. The path assembly step (step 3) takes between 3 and 9 CPU hours. Finally, the refinement step (step 4) takes between 4 and 6 CPU hours per model. LZerD is available for download at http://www.kiharalab.org/proteindocking/lzerd.php. IDP-LZerD is available for download at http://www.kiharalab.org/proteindocking/idp_lzerd.tar.bz2

## Supporting information

S1 Fig The minimum RMSD of the fragments generated by Rosetta Fragment Picker for each window of 1devB. Results for each of the six windows of 1devB are plotted in different colors. Blue: window 1; green: 2; red: 3; purple: 4; yellow: 5; cyan: 6. The RMSD is computed using all atoms. The minimum RMSD decreases only modestly as more fragments are picked.
(TIF)

S2 Fig Reduction of the search space by the pre-filtering and clustering procedures. The x-axis shows the stage of path assembly and the y-axis shows the total number of paths remaining to consider. The number of possible paths was reduced by pre-filtering improbable pairs of docked fragments (Fig 8) and clustering paths. The maximum number of paths is 4500^*N*^, where 4,500 is the number of docked fragments for a window and *N* is the number of windows. 2P shows the number of 2-window pairs that were not pre-filtered multiplied by the remaining possible combinations (4500^*N*−2^). 2C shows the number of 2-window cluster centers multiplied by the remaining possible combinations, and so on. Thus, the decrease in possible paths from Max to 2P is due to pre-filtering while the decrease from 2P to 2C is due to clustering. Data from six targets, 1l2w, 1bk5, 1l3e, 1axc, 4ah2, and 1ycr, are shown.
(TIF)

S3 Fig Results of the structure refinement. **(A)**: C_*α*_ distances of neighboring residues before (red) and after (blue) refinement. Bars are in purple when red and blue bars overlap. Data taken from rank 1 models of all training complexes. **(B)**: Change in L-RMSD (Å) due to refinement. Data from all training complexes.
(TIF)

S4 Fig Using BindML binding site residue prediction for model pre-filtering. **(A)**: Effect of BindML score cutoff values on prediction accuracy. In BindML, a confidence score is provided for each predicted binding site residue, with a smaller (more negative) value more confident. Blue: precision; green: recall; red: *F*_1_-score. Vertical lines show 95% confidence interval of the mean. Prediction results are taken from all bound training complexes. The plot shows that the *F*_1_-score of the BindML prediction increased as the cutoff became more permissive since recall increased dramatically while precision stayed at almost the same level. Residues with a BindML Z-score ≤ −0.25 were considered as interface. **(B)**: L-RMSD of models relative to the agreement of predicted interface residues and model interface residues. For models of a target (after step 4), the fraction of BindML predicted receptor interface residues that are located at the interface in the model (*f*_BindML_) was computed. Then, the models were sorted by the Z-score of this fraction among all the paths of the target (*Z*(*f*_BindML_)). In the model selection using BindML prediction, paths that have a Z-score of 1.5 or larger were selected as a pre-filtering step. The panels show examples of correlation between *Z*(*f*_BindML_) and L-RMSD. A weak inverse correlation was observed for 1p4qBA (left) and 1sqkAB (center) but the procedure did not work for 1ipbAB (right).
(TIF)

S5 Fig Effect of secondary structure accuracy on overall accuracy. X-axis: secondary structure for a residue in the native structure and the model; e.g. “HC” means the native structure is helix and the model is coil. Y-axis: C_*α*_ RMSD of the residue. Star (*) indicates the mean. Group means are significantly different by one-way ANOVA (*p* = 1 × 10^−35^). Using Tukey’s range test, C_*α*_ RMSD is significantly lower for HH than HC, CH, and CC and C_*α*_ RMSD is significantly lower for HC than CC and CH. Secondary structure computed using DSSP [48]. DSSP classes GHI are considered H, EB are considered E, and all others are considered C. We did not include bars with E because only 12 residues were classified as E. Computed using the top 10 models of 1ycrB, 1fv1C, 1wkwB, 2cpkI, 1sb0B, 1sqkB, 2bzwB, 3owtC, 1devB, 1l8cB, and 1xtgB.
(TIF)

S6 Fig Effect of secondary structure prediction agreement on overall accuracy. X-axis: the number of methods that predict the secondary structure class shown in the native. Y-axis: C_*α*_ RMSD of the residues. Star (*) indicates the mean. Group means are significantly different by one-way ANOVA (*p* = 1 × 10^−11^) Using Tukey’s range test, C_*α*_ RMSD is significantly higher for 0 than 2, 3, and 4. Computed using the top 10 models of 1ycrB, 1fv1C, 1wkwB, 2cpkI, 1sb0B, 1sqkB, 2bzwB, 3owtC, 1devB, 1l8cB, and 1xtgB.
(TIF)

S7 Fig Frequency of IDP contacts between TCF7L2 and *β*-catenin. For each plot, the x-axis lists all residues in the IDP and the y-axis shows the number of models in the final 1000 where that IDP residue is in contact with the receptor (5 Å cutoff distance). Gray bars indicate experimentally verified hotspot residues. Horizontal line shows the mean number of models. Top: bound (1jpw); bottom: unbound (2z6h).
(TIF)

S8 Fig Frequency of IDP contacts between CITED2 and p300. For each plot, the x-axis lists all residues in the IDP and the y-axis shows the number of models in the final 1000 where that IDP residue is in contact with the receptor (5 Å cutoff distance). Gray bars indicate experimentally verified hotspot residues. Horizontal line shows the mean number of models. Top: bound (1p4q); bottom: cross-docking (1l3e).
(TIF)

S9 Fig Frequency of IDP contacts between HIF-1*α* and p300. For each plot, the x-axis lists all residues in the IDP and the y-axis shows the number of models in the final 1000 where that IDP residue is in contact with the receptor (5 Å cutoff distance). Gray bars indicate experimentally verified hotspot residues. Horizontal line shows the mean number of models. Top: bound (1l8c); bottom: unbound (1u2n).
(TIF)

S10 Fig Distance distributions observed in structures annotated as disordered in DisProt. These were used to heuristically determine distance cutoffs for pairs of docked fragments (Fig 8). Vertical lines indicate minimum and maximum allowed values for the color-matched distribution. Top: distances corresponding to neighboring sequence windows. Separation 4 (green) is overlap atom distance, 5.2 Å ≤ *d* ≤ 13.6 Å. Separation 6 (blue) is midpoint residue distance, 6.5 Å ≤ *d* ≤ 18.5 Å. Bottom: distances corresponding to non-neighboring sequence windows. Separation 12 (purple) is the midpoint residue distance for a window separation of 2 (i.e. window A and C), 3.8 Å ≤ *d* ≤ 37 Å. Separation 18 (red) is the midpoint residue distance for a window separation of 3 (i.e. A and D), 3.8 Å ≤ *d* ≤ 55.5 Å.
(TIF)

S1 Table Accuracy cutoffs used by CAPRI. (PDF)

S2 Table Minimum RMSD at each step of modeling. (PDF)

S3 Table Scoring function performance on docked fragments. (PDF)

S4 Table Scoring function performance on selecting paths. (PDF)

S5 Table Scoring function performance on relaxed models. (PDF)

S6 Table MD-based protein-peptide docking test set. (PDF)

S7 Table 2-fold cross validation for optimizing Path and Model Scores. (PDF)
