# Supplementary figures and images for: Modeling disordered protein interactions from biophysical principles
Source: PLoS Comput Biol. 2017 Apr 10;13(4):e1005485. doi: 10.1371/journal.pcbi.1005485 (PMC5402988; doi:10.1371/journal.pcbi.1005485)

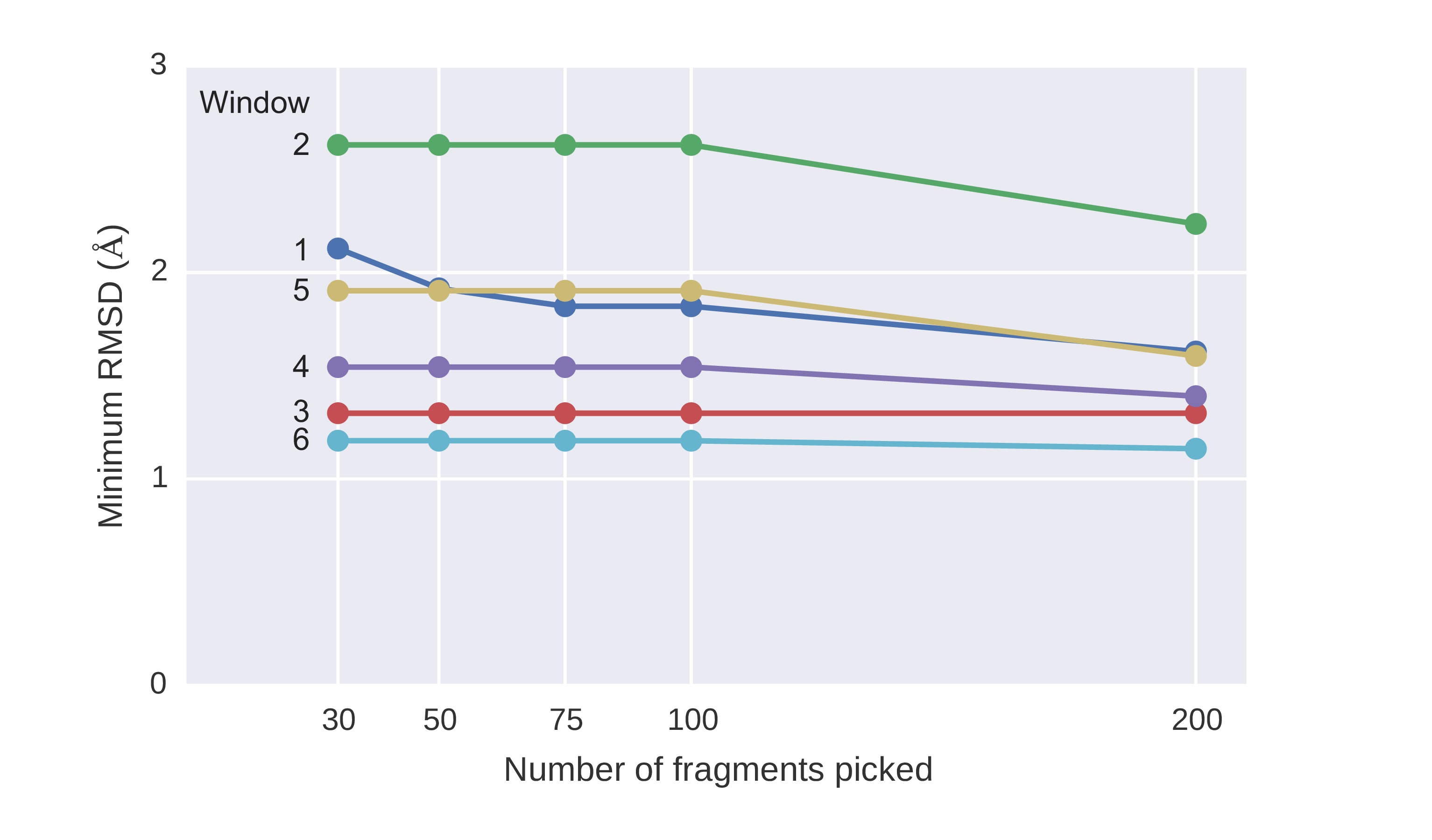

Supplement: S1 Fig — Results for each of the six windows of 1devB are plotted in different colors. Blue: window 1; green: 2; red: 3; purple: 4; yellow: 5; cyan: 6. The RMSD is computed using all atoms. The minimum RMSD decreases only modestly as more fragments are picked. (TIF) [file pcbi.1005485.s001.tif]

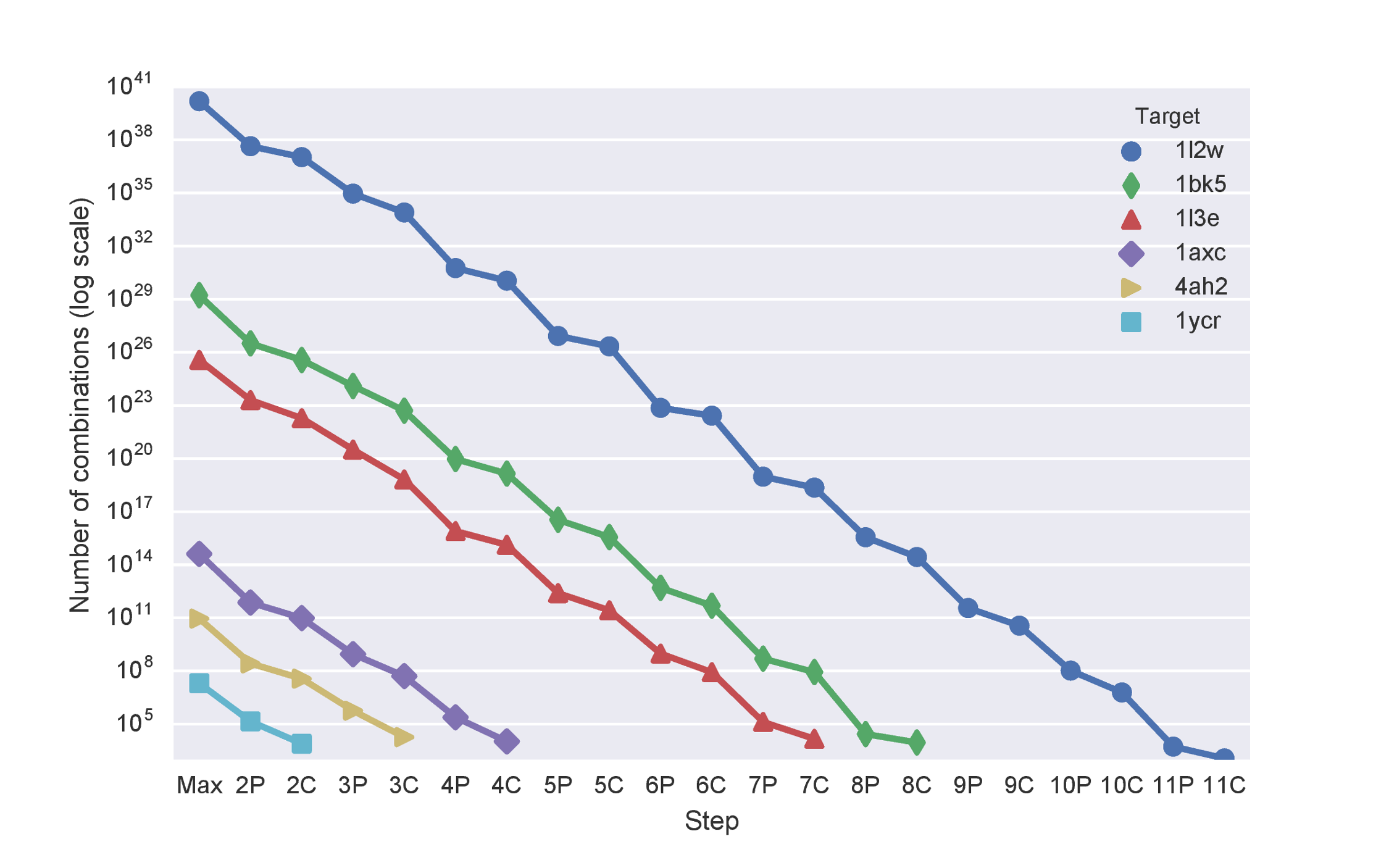

Supplement: S2 Fig — The x-axis shows the stage of path assembly and the y-axis shows the total number of paths remaining to consider. The number of possible paths was reduced by pre-filtering improbable pairs of docked fragments (Fig 8) and clustering paths. The maximum number of paths is 4500N, where 4,500 is the number of docked fragments for a window and N is the number of windows. 2P shows the number of 2-window pairs that were not pre-filtered multiplied by the remaining possible combinations (4500N−2). 2C shows the number of 2-window cluster centers multiplied by the remaining possible combinations, and so on. Thus, the decrease in possible paths from Max to 2P is due to pre-filtering while the decrease from 2P to 2C is due to clustering. Data from six targets, 1l2w, 1bk5, 1l3e, 1axc, 4ah2, and 1ycr, are shown. (TIF) [file pcbi.1005485.s002.tif]

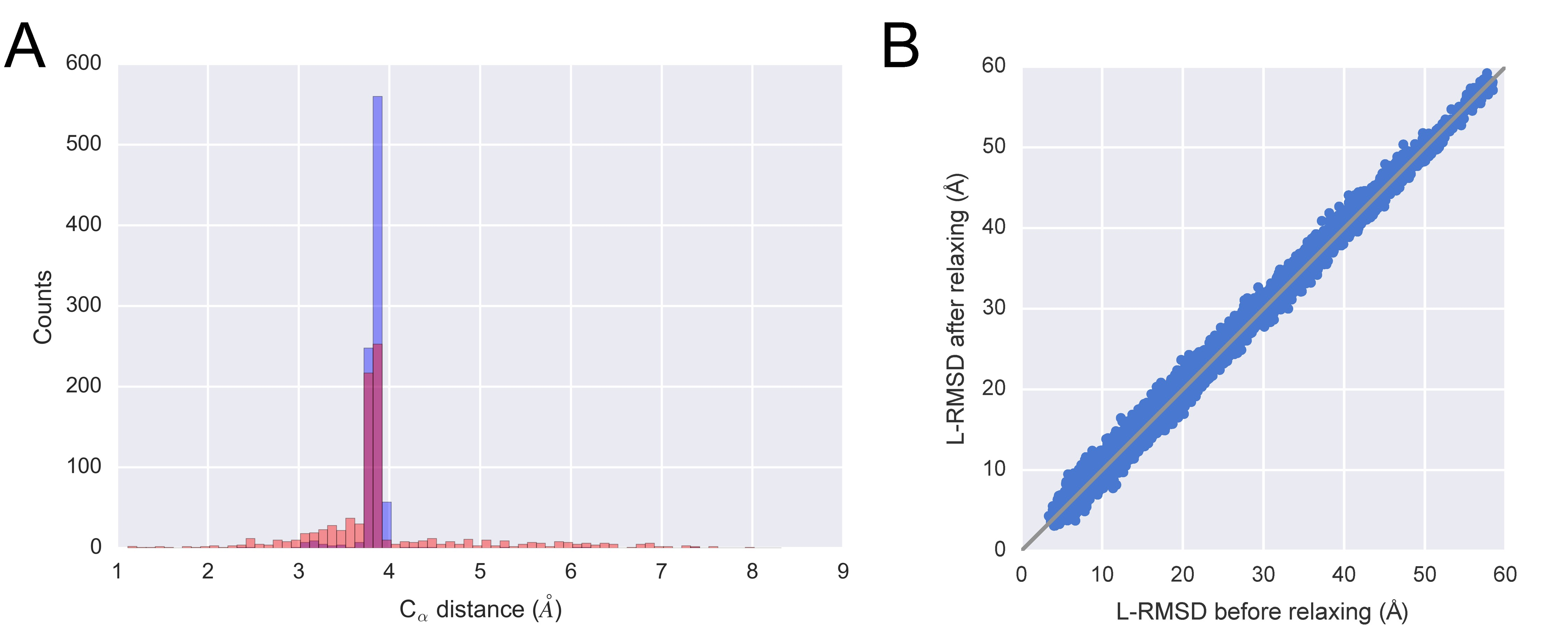

Supplement: S3 Fig — (A): Cα distances of neighboring residues before (red) and after (blue) refinement. Bars are in purple when red and blue bars overlap. Data taken from rank 1 models of all training complexes. (B): Change in L-RMSD (Å) due to refinement. Data from all training complexes. (TIF) [file pcbi.1005485.s003.tif]

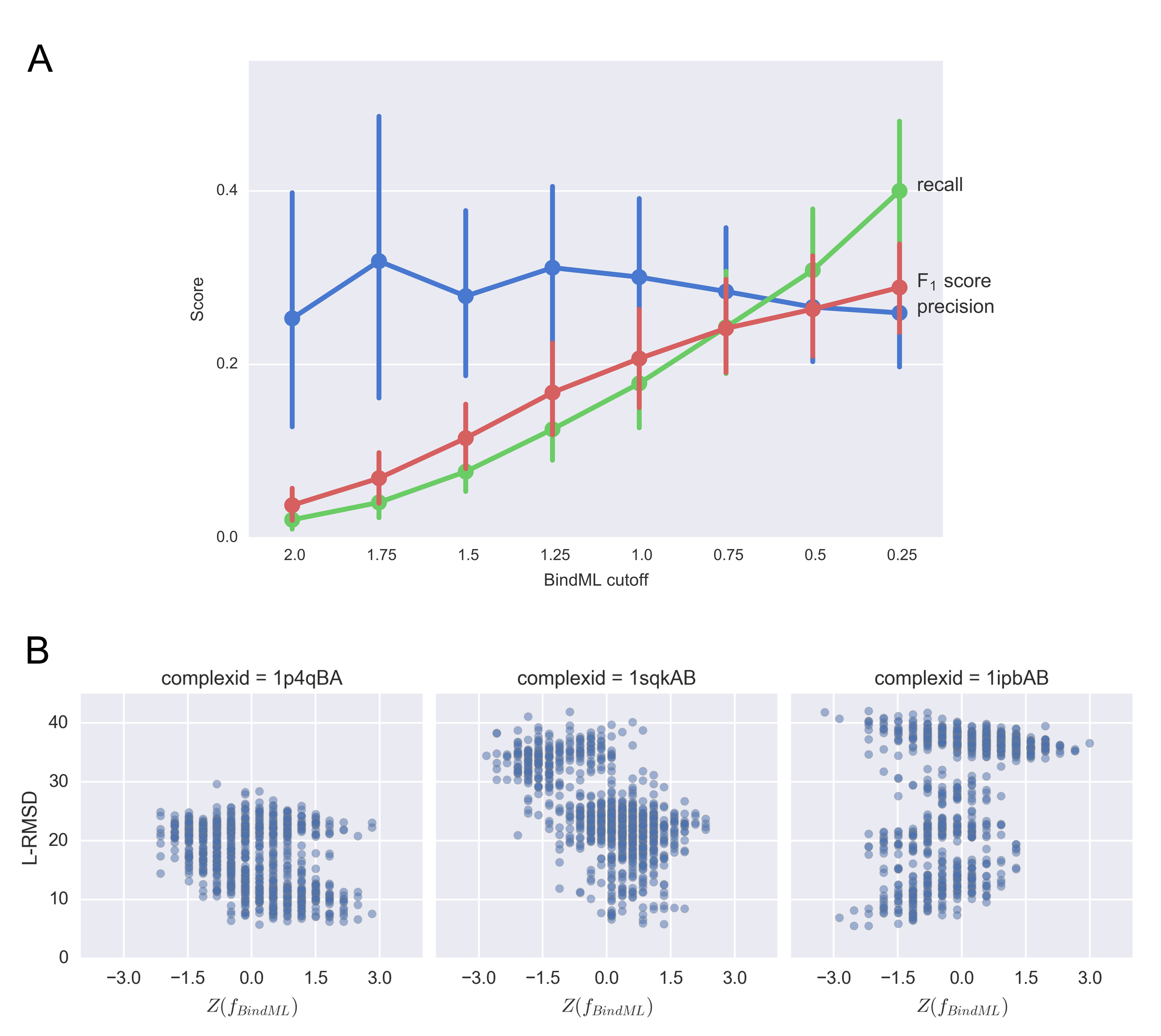

Supplement: S4 Fig — (A): Effect of BindML score cutoff values on prediction accuracy. In BindML, a confidence score is provided for each predicted binding site residue, with a smaller (more negative) value more confident. Blue: precision; green: recall; red: F1-score. Vertical lines show 95% confidence interval of the mean. Prediction results are taken from all bound training complexes. The plot shows that the F1-score of the BindML prediction increased as the cutoff became more permissive since recall increased dramatically while precision stayed at almost the same level. Residues with a BindML Z-score ≤ −0.25 were considered as interface. (B): L-RMSD of models relative to the agreement of predicted interface residues and model interface residues. For models of a target (after step 4), the fraction of BindML predicted receptor interface residues that are located at the interface in the model (fBindML) was computed. Then, the models were sorted by the Z-score of this fraction among all the paths of the target (Z(fBindML)). In the model selection using BindML prediction, paths that have a Z-score of 1.5 or larger were selected as a pre-filtering step. The panels show examples of correlation between Z(fBindML) and L-RMSD. A weak inverse correlation was observed for 1p4qBA (left) and 1sqkAB (center) but the procedure did not work for 1ipbAB (right). (TIF) [file pcbi.1005485.s004.tif]

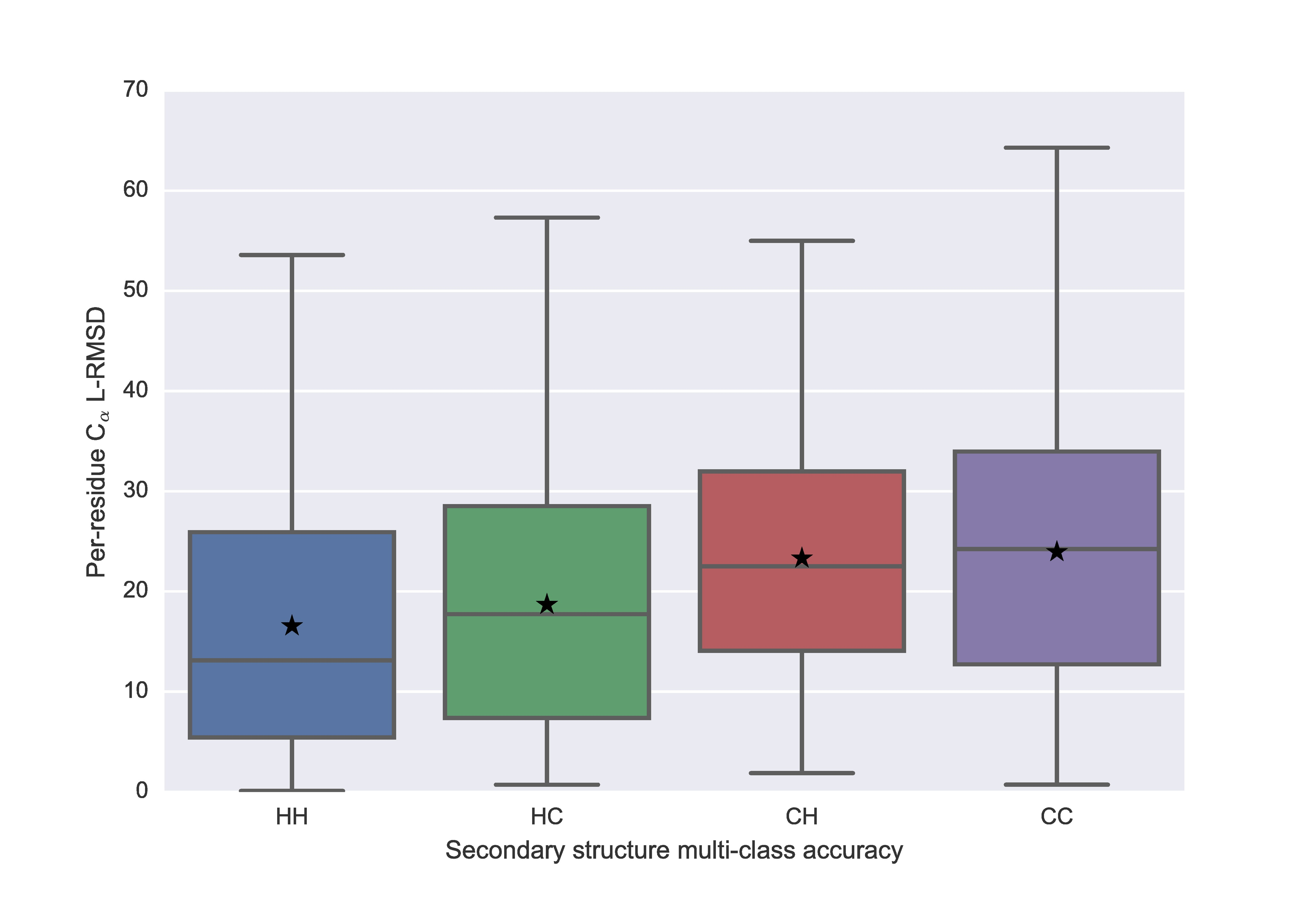

Supplement: S5 Fig — X-axis: secondary structure for a residue in the native structure and the model; e.g. “HC” means the native structure is helix and the model is coil. Y-axis: Cα RMSD of the residue. Star (*) indicates the mean. Group means are significantly different by one-way ANOVA (p = 1 × 10^−35^). Using Tukey’s range test, Cα RMSD is significantly lower for HH than HC, CH, and CC and Cα RMSD is significantly lower for HC than CC and CH. Secondary structure computed using DSSP [48]. DSSP classes GHI are considered H, EB are considered E, and all others are considered C. We did not include bars with E because only 12 residues were classified as E. Computed using the top 10 models of 1ycrB, 1fv1C, 1wkwB, 2cpkI, 1sb0B, 1sqkB, 2bzwB, 3owtC, 1devB, 1l8cB, and 1xtgB. (TIF) [file pcbi.1005485.s005.tif]

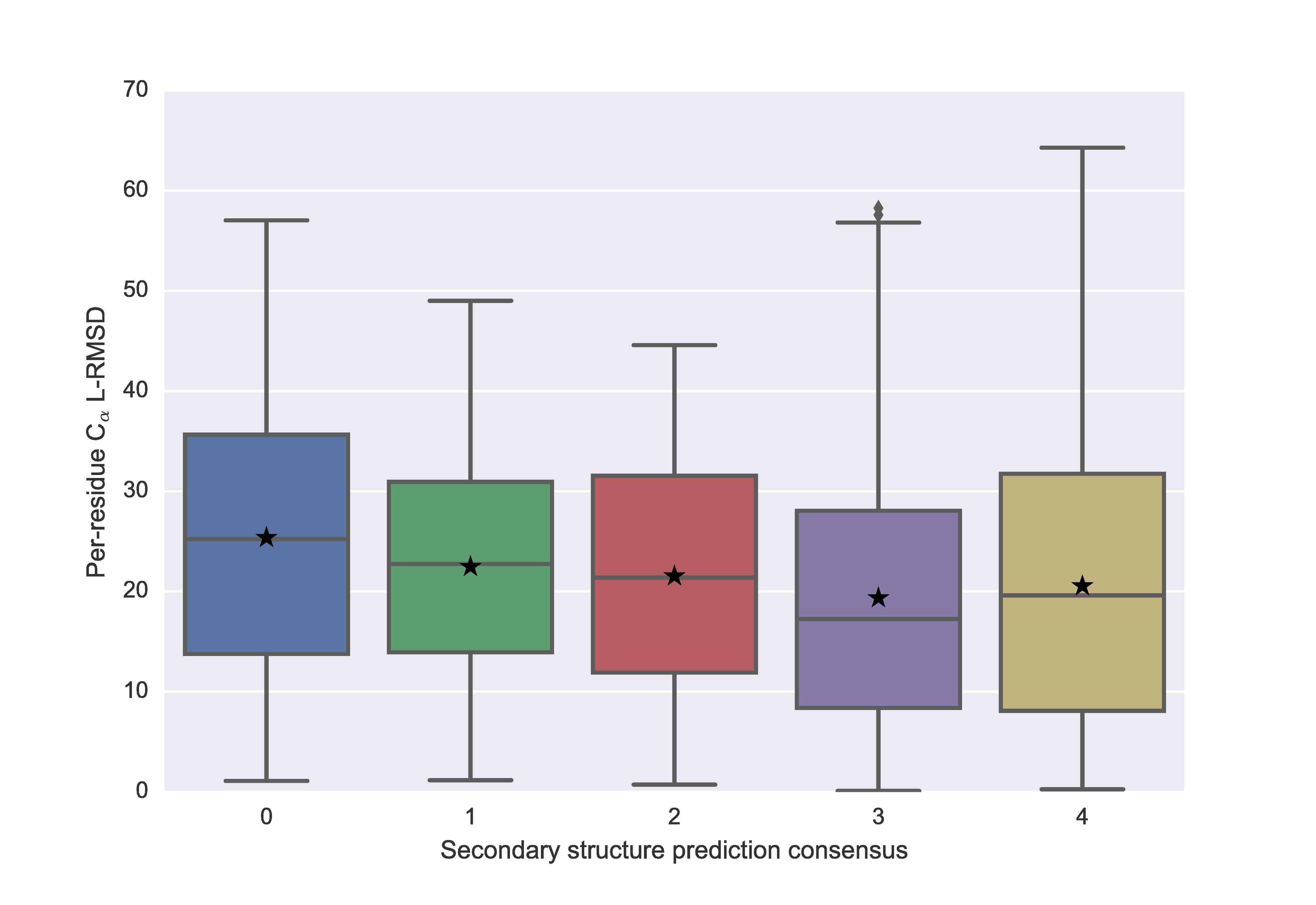

Supplement: S6 Fig — X-axis: the number of methods that predict the secondary structure class shown in the native. Y-axis: Cα RMSD of the residues. Star (*) indicates the mean. Group means are significantly different by one-way ANOVA (p = 1 × 10^−11^) Using Tukey’s range test, Cα RMSD is significantly higher for 0 than 2, 3, and 4. Computed using the top 10 models of 1ycrB, 1fv1C, 1wkwB, 2cpkI, 1sb0B, 1sqkB, 2bzwB, 3owtC, 1devB, 1l8cB, and 1xtgB. (TIF) [file pcbi.1005485.s006.tif]

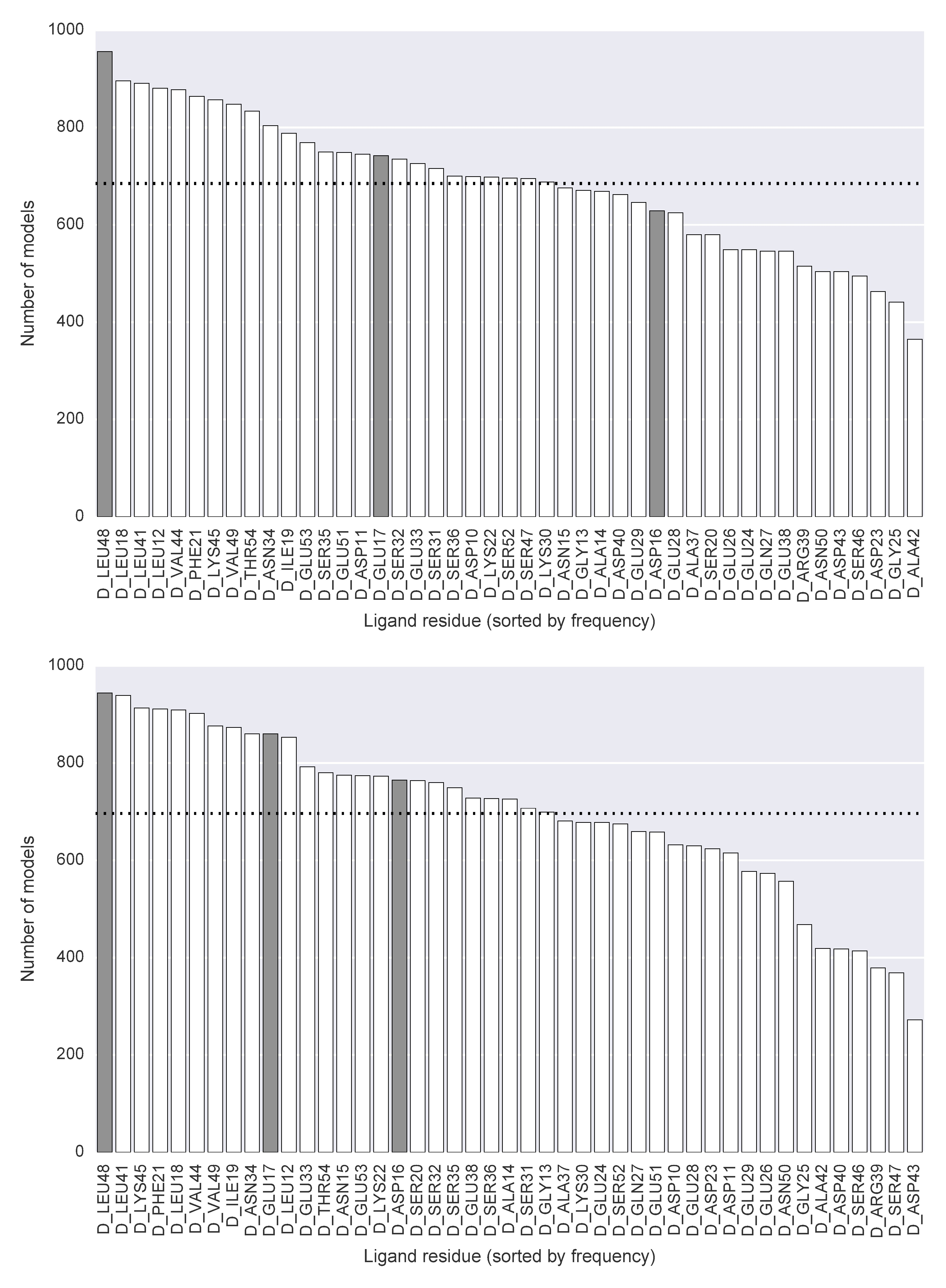

Supplement: S7 Fig — For each plot, the x-axis lists all residues in the IDP and the y-axis shows the number of models in the final 1000 where that IDP residue is in contact with the receptor (5 Å cutoff distance). Gray bars indicate experimentally verified hotspot residues. Horizontal line shows the mean number of models. Top: bound (1jpw); bottom: unbound (2z6h). (TIF) [file pcbi.1005485.s007.tif]

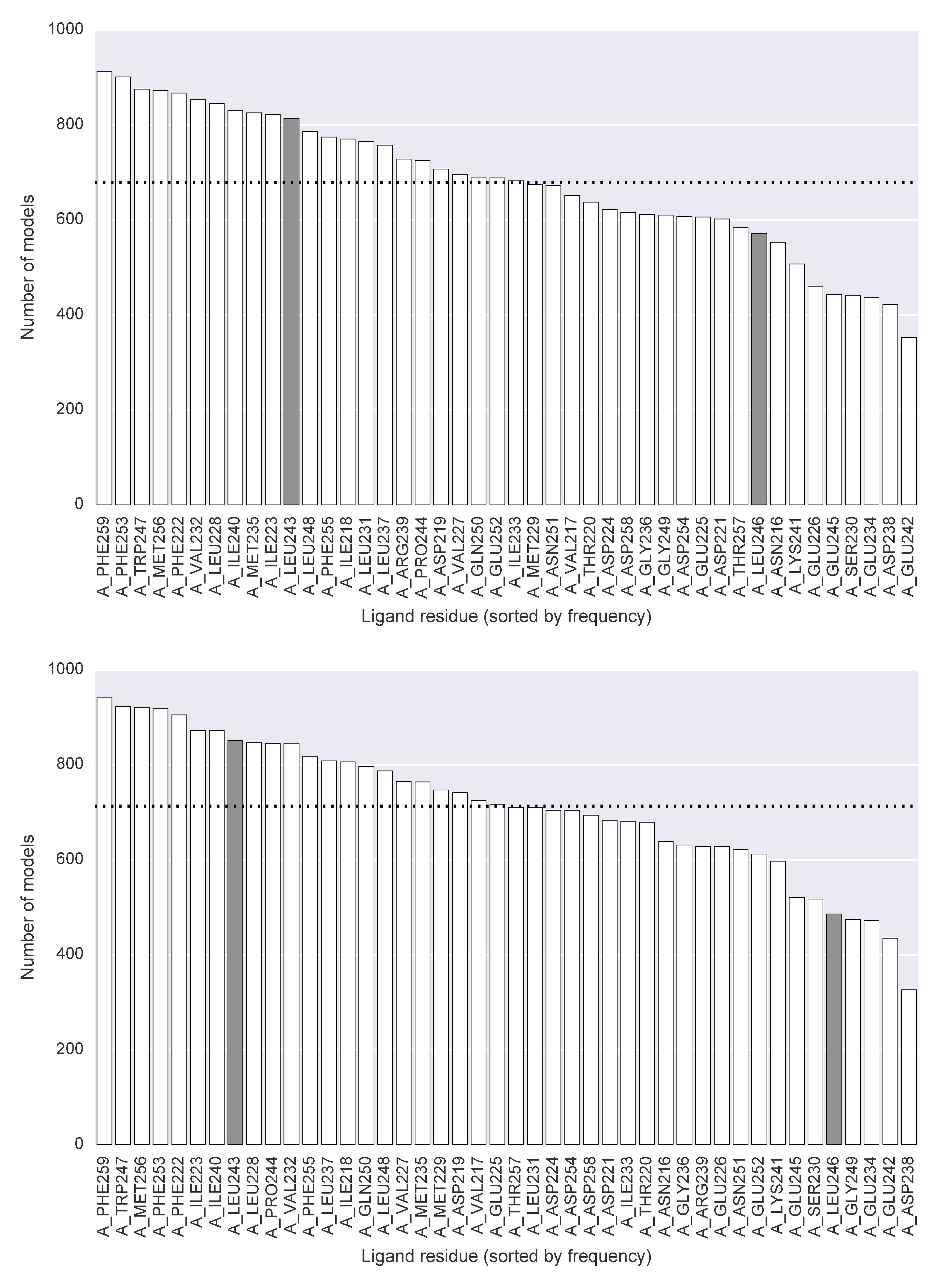

Supplement: S8 Fig — For each plot, the x-axis lists all residues in the IDP and the y-axis shows the number of models in the final 1000 where that IDP residue is in contact with the receptor (5 Å cutoff distance). Gray bars indicate experimentally verified hotspot residues. Horizontal line shows the mean number of models. Top: bound (1p4q); bottom: cross-docking (1l3e). (TIF) [file pcbi.1005485.s008.tif]

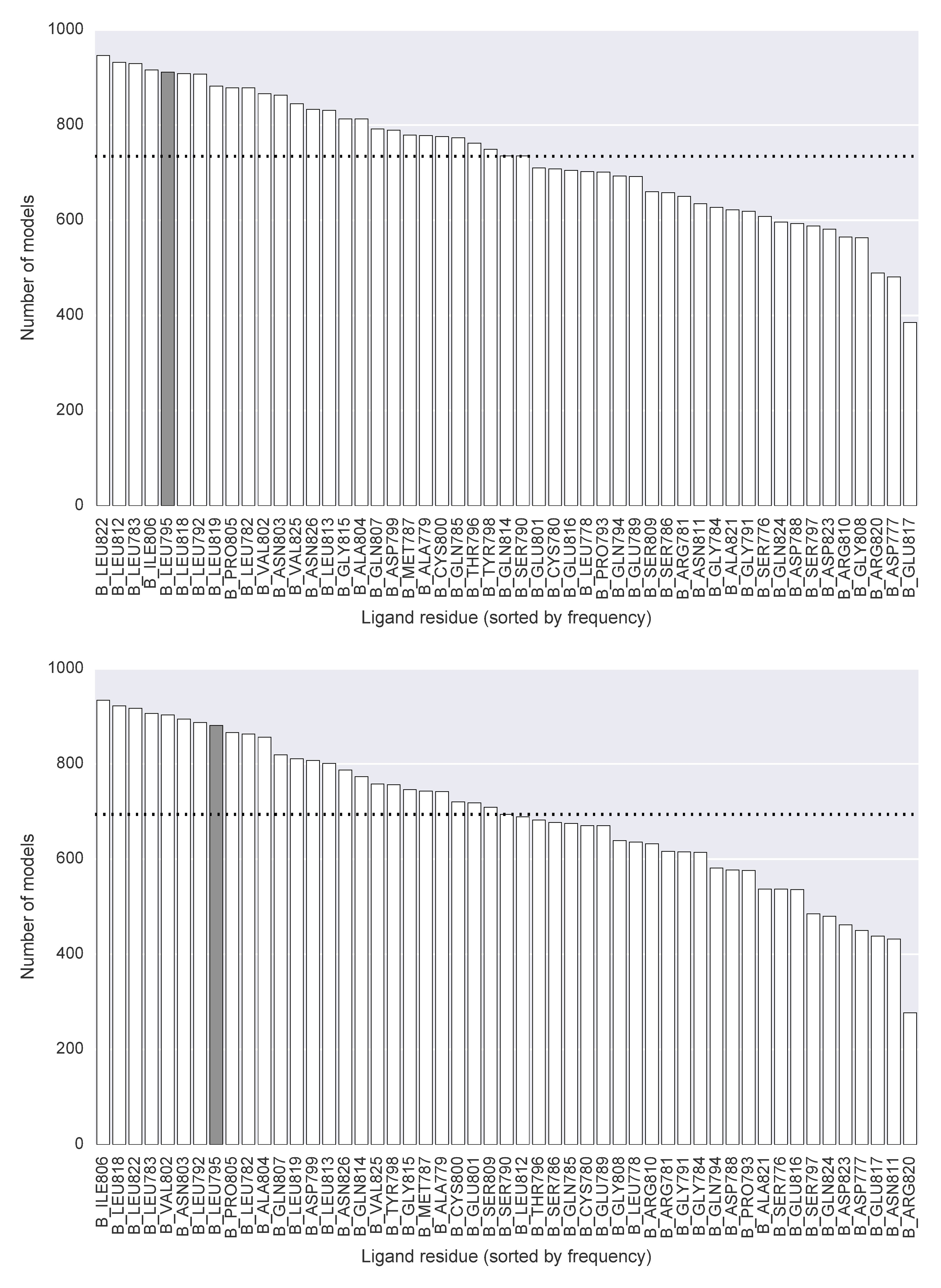

Supplement: S9 Fig — For each plot, the x-axis lists all residues in the IDP and the y-axis shows the number of models in the final 1000 where that IDP residue is in contact with the receptor (5 Å cutoff distance). Gray bars indicate experimentally verified hotspot residues. Horizontal line shows the mean number of models. Top: bound (1l8c); bottom: unbound (1u2n). (TIF) [file pcbi.1005485.s009.tif]

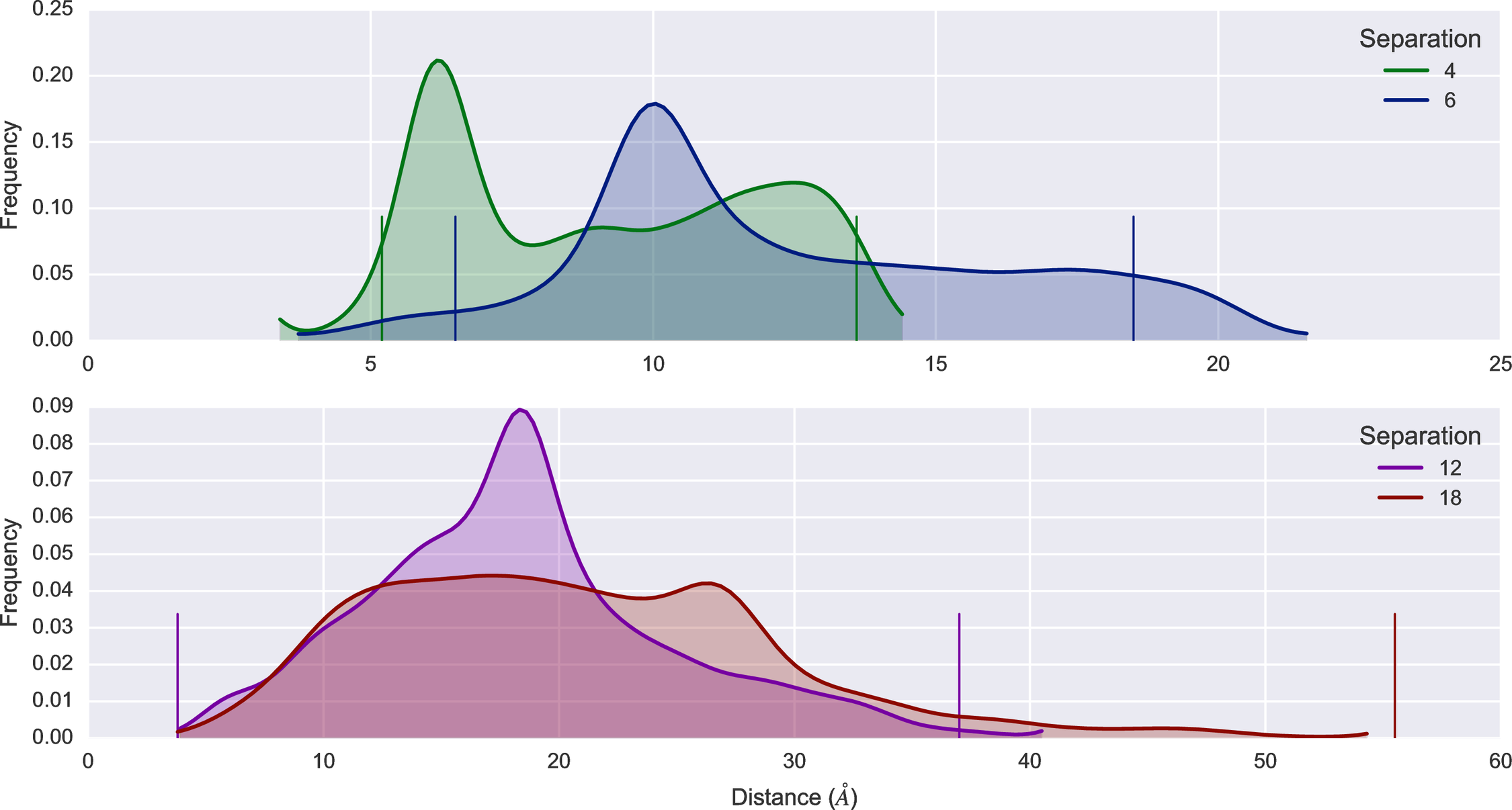

Supplement: S10 Fig — These were used to heuristically determine distance cutoffs for pairs of docked fragments (Fig 8). Vertical lines indicate minimum and maximum allowed values for the color-matched distribution. Top: distances corresponding to neighboring sequence windows. Separation 4 (green) is overlap atom distance, 5.2 Å ≤ d ≤ 13.6 Å. Separation 6 (blue) is midpoint residue distance, 6.5 Å ≤ d ≤ 18.5 Å. Bottom: distances corresponding to non-neighboring sequence windows. Separation 12 (purple) is the midpoint residue distance for a window separation of 2 (i.e. window A and C), 3.8 Å ≤ d ≤ 37 Å. Separation 18 (red) is the midpoint residue distance for a window separation of 3 (i.e. A and D), 3.8 Å ≤ d ≤ 55.5 Å. (TIF) [file pcbi.1005485.s010.tif]
