# Supplementary material for: Modeling disordered protein interactions from biophysical principles
Source: PLoS Comput Biol. 2017 Apr 10;13(4):e1005485. doi: 10.1371/journal.pcbi.1005485 (PMC5402988; doi:10.1371/journal.pcbi.1005485)
Supplement: S1 Table — (PDF) [file pcbi.1005485.s011.pdf]

S1 Table: Accuracy cutoffs used by CAPRI

Quality	f_{nat}	L-RMSD		I-RMSD
High	≥ 0.5	≤ 1.0	or	≤ 1.0
Medium	≥ 0.3	$1.0 < x \leq 5.0$	or	$1.0 < x \leq 2.0$
Acceptable	≥ 0.1	$5.0 < x \leq 10.0$	or	$2.0 < x \leq 4.0$
Incorrect	< 0.1			

The four quality levels of docking models used in the Critical Assessment of Predicted Interactions (CAPRI) and their criteria. f_{nat}: the fraction of native contacts that are reproduced in a predicted docking model. Native contacts are defined as pairs of residues from interacting proteins with any heavy atoms within 5.0 Å of each other. L-RMSD: Ligand RMSD, the backbone (N, C_α, C, O) RMSD of the ligand (here docked IDP) after superimposition of a docking model to its native complex structure using the receptor structure. I-RMSD: Interface RMSD, the backbone (N, C_α, C, O) RMSD of the interface residues. An interface residue is defined as having any heavy atom within 10.0 Å of any heavy atom in a docked partner protein. Adapted from [39].
