# Supplementary material for: Modeling disordered protein interactions from biophysical principles
Source: PLoS Comput Biol. 2017 Apr 10;13(4):e1005485. doi: 10.1371/journal.pcbi.1005485 (PMC5402988; doi:10.1371/journal.pcbi.1005485)
Supplement: S2 Table — (PDF) [file pcbi.1005485.s012.pdf]

S2 Table: Minimum RMSD at each step of modeling

Bound	Steps 1-2: Frag. Str. Pred. & Docking Max Min RMSD (Å)			Step 3: Combining Docked Frag. Min RMSD (Å)			Unbound	Step 2: Frag. Docking Max Min RMSD (Å)		Step 3: Combining Docked Frag. Min RMSD (Å)		
Receptor PDB ID	Fragments	All docked	Selected docked	All paths	Clustered paths	Selected paths	Receptor PDB ID	All docked	Selected docked	All Paths	Clustered paths	Selected paths
1ycrA	1.4	3.1	3.2	3.1	3.6	3.6	1z1mA	4.2	4.2	4.1	4.4	4.4
1fv1AB	1.3	3.9	5.3	4.1	4.8	5.0	4ah2AB	4.7	4.9	4.4	5.0	5.4
1wkwA	1.5	3.3	3.9	4.2	4.9	5.5	1ipbA	3.0	4.5	4.7	5.3	5.3
2cpkE	2.6	3.4	3.6	3.8	4.2	4.9	1j3hA	3.7	4.3	3.4	4.1	5.5
1sb0A	1.8	5.2	7.4	4.9	4.9	5.3	4i9oA	6.9	7.7	6.3	6.3	7.5
1sqkA	2.0	3.3	8.1	5.4	5.4	5.8	1ijjA	3.4	7.4	5.2	5.2	5.2
2bzwA	0.8	2.5	5.0	4.5	4.9	4.9	1pq0A	4.3	6.4	11.0	11.3	12.0
3owtAB	1.1	2.9	4.3	3.4	3.5	5.4	3cz6AB	3.2	4.7	4.3	4.4	6.4
1devA	2.2	3.5	4.7	6.4	6.6	6.9	1khxA	3.6	7.1	7.1	7.4	9.4
1p4qB	2.2	4.4	5.1	4.9	5.3	6.5	1l3eB	3.8	4.9	5.3	5.5	6.1
1jpwA	1.4	3.7	3.9	4.5	4.5	4.8	2z6hA	6.9	11.3	7.5	7.5	7.7
1l8cA	2.3	4.0	5.1	4.8	5.1	5.3	1u2nA	5.4	5.4	5.8	6.1	6.2
2c1tA	1.4	4.1	10.4	20.8	20.8	22.0	1bk5A	3.7	12.0	17.7	17.7	19.5
1xtgA	3.4	5.3	5.6	8.4	8.4	8.4	1xtfA	5.4	6.5	11.1	11.4	11.4
Train Avg	1.8	3.7	5.4	5.9	6.2	6.7		4.4	6.5	7.0	7.3	8.0
2w84A	0.4	3.3	3.5	3.4	4.4	4.4	5aonA	3.2	3.2	3.8	4.1	4.3
1axcA	2.6	3.9	6.6	6.5	8.6	8.9	1vymA	3.8	6.0	5.4	6.0	10.2
2pheAB	1.7	3.5	4.1	5.1	5.3	7.1	1pcfAB	4.3	4.9	7.1	7.2	7.9
1g0vA	0.4	2.2	2.2	3.6	3.9	3.9	1fmxA	2.8	3.8	4.1	4.2	4.2
3wn7A	1.4	3.5	7.6	7.5	7.5	7.5	1x2jA	3.8	7.6	8.3	8.3	8.3
1s70A	2.4	3.7	6.4	7.8	8.2	8.7	4ut2A	3.7	8.5	10.2	10.3	13.7
2o8gA	1.7	8.2	9.0	7.9	8.0	8.5	1jk7A	8.7	9.9	8.4	8.5	8.5
1l2wAB	2.5	4.3	6.6	10.6	10.6	10.6	1jyaAB	4.3	6.2	8.7	8.8	8.8
Test Avg	1.6	4.1	5.7	6.6	7.1	7.5		4.3	6.3	7.0	7.2	8.2
All Avg	1.8	3.9	5.5	6.2	6.5	7.0		4.4	6.4	7.0	7.2	8.1

Step 1 of the modeling predicts fragment structures of each sequence window of an IDP. Max Min RMSD: the minimum RMSD is calculated for each window and the maximum (i.e. worst) window-minimum RMSD is shown for each complex. Fragment: backbone RMSD of predicted fragments against native. Values are not shown for the unbound receptors on the right side of the table because the same fragment structures were used for an IDP for both bound and unbound receptors.

Step 2 of the modeling docks each fragment to the bound and unbound receptors. All docked: L-RMSD of docked fragments (has a lower bound of Fragment RMSD). Selected docked: 4,500 fragments for each window selected by DI score were considered.

Step 3 of the modeling is to combine fragments into paths of the full length IDPs. All path: the minimum RMSD of all generated paths. Clustered path: the minimum RMSD of cluster centers of paths with a cutoff of a 4.0 Å RMSD. Selected path: the minimum RMSD of 1000 path cluster centers selected by Path Score.
