# Supplementary material for: Modeling disordered protein interactions from biophysical principles
Source: PLoS Comput Biol. 2017 Apr 10;13(4):e1005485. doi: 10.1371/journal.pcbi.1005485 (PMC5402988; doi:10.1371/journal.pcbi.1005485)
Supplement: S3 Table — (PDF) [file pcbi.1005485.s013.pdf]

S3 Table: Scoring function performance on docked fragments

Score	Average			Losses		
	EF5	EF10	min RMSD	EF5	EF10	min RMSD
DFIRE	2.56	1.62	3.77	4	10	4
GOAP	2.00	1.61	3.75	9	6	7
ITScorePro	2.11	1.40	3.41	3	5	2
DI Score	2.47	1.58	3.48	0	0	0

To select docked fragments for combination in Step 3, three single scoring terms and one combination were evaluated.

EF5/EF10: the enrichment factor of docked fragments with L-RMSD $\leq$ 5/10 Å, averaged across all windows. Enrichment factor is the ratio between the percentage of hits in the chosen pool and the percentage of hits in the entire pool; a value above 1.0 indicates that the score has enriched the pool. Min RMSD: the minimum L-RMSD of a selected docked fragment, averaged across all windows. Losses: for each window, the performance of each scoring function was ranked, and the number of windows where the scoring function ranked lowest was counted. Values are calculated using the top 4500 docked fragments from each window by the score using 1devAB, 1l2wABI, and 2cpkEI.

On average, DFIRE had the highest (best) EF5 and EF10 while ITScorePro had the lowest (best) RMSD. However, when comparing scores within a target window, DFIRE was most frequently ranked last for EF10 while ITScorePro was rarely ranked last. This indicates that DFIRE had excellent selection performance on many targets but ITScorePro was more consistent across targets. DI Score, the sum of the Z-scores of DFIRE and ITScorePro, balances the performance of DFIRE and the consistency of ITScorePro. DI Score had comparable performance for EF5, EF10, and min RMSD and was never ranked last.
