# Supplementary material for: Modeling disordered protein interactions from biophysical principles
Source: PLoS Comput Biol. 2017 Apr 10;13(4):e1005485. doi: 10.1371/journal.pcbi.1005485 (PMC5402988; doi:10.1371/journal.pcbi.1005485)
Supplement: S4 Table — (PDF) [file pcbi.1005485.s014.pdf]

S4 Table: Scoring function performance on selecting paths

Score	Minimum recall (%)
Energy score (S_E)	0.77
Overlap score (S_O)	0.64
Cluster size (S_C)	0
Receptor score (S_R)	0
Min. Z-score	2.0
Linear combination 4	2.7
Linear combination 5 (Path Score)	2.8
Random	0.95

To select paths for refinement in Step 4, four single scoring terms and two linear combinations were evaluated.

Min. Z-score: for each path, the lowest Z-score among S_E, S_O, S_C, and S_R; Linear combination 4: Weights on S_E, S_O, S_C, and S_R (w_1-w_4 in Eqn 1); Linear combination 5: Weights on “Min. Z-score” and “Linear combination 4” (w_5 in Eqn 1). The minimum recall across all complexes is shown; recall is the number of hits retrieved by a given score divided by the total number of hits. A path is considered a hit if it has pooled RMSD $\leq$ 10 Å. Values are calculated using the top 1000 paths by the scoring function. Random recall is 1000 divided by the total number of paths. Weights were trained using 1devAB, 1fv1ABC, 1ijjAB, 1ipbAB, 1j3hAI, 1jpwAD, 1khxAB, 1l8cAB, 1p4qBA, 1pq0AB, 1sb0AB, 1sqkAB, 1u2nAB, 1wkwAB, 1xtfAB, 1xtgAB, 1ycrAB, 2bzwAB, 2c1tAC, 2cpkEI, 2z6hAD, and 3owtABC. Values are reported for these training complexes.
