# Supplementary material for: Modeling disordered protein interactions from biophysical principles
Source: PLoS Comput Biol. 2017 Apr 10;13(4):e1005485. doi: 10.1371/journal.pcbi.1005485 (PMC5402988; doi:10.1371/journal.pcbi.1005485)
Supplement: S5 Table — (PDF) [file pcbi.1005485.s015.pdf]

S5 Table: Scoring function performance on relaxed models

Score	Mean RFH	Complexes with RFH in top: 1	10	100
DFIRE	36.7	4	8	16
GOAP	16.4	4	9	18
ITScorePro	23.3	2	12	17
Molecular mechanics	28.9	4	9	16
Min. Z-score	15.0	3	9	18
Linear combination 4	12.0	3	11	18
Linear combination 5 (Model Score)	11.3	3	11	18
Random	38.6	0	7	16

To re-rank models after refinement in Step 4, four single scoring terms and two linear combinations were evaluated.

RFH: numerical rank of the first hit (good model); Min. Z-score: for each path, the lowest Z-score among ITScorePro, DFIRE, molecular mechanics score, and GOAP; Linear combination 4: Weights on ITScorePro, DFIRE, molecular mechanics score, and GOAP (w_1-w_4 in Eqn 4); Linear combination 5: Weights on "Min. Z-score" and "Trained 4" (w_5 in Eqn 4). A path is considered a hit if it meets CAPRI "Acceptable" criteria (S1 Table). Random values were estimated by shuffling the models 1000 times. Weights were trained using 1devAB, 1fv1ABC, 1ijjAB, 1ipbAB, 1j3hAI, 1jpwAD, 1khxAB, 1l8cAB, 1p4qBA, 1pq0AB, 1sb0AB, 1sqkAB, 1u2nAB, 1wkwAB, 1xtfAB, 1xtgAB, 1ycrAB, 2bzwAB, 2c1tAC, 2cpkEI, 2z6hAD, and 3owtABC. Values are reported for these training complexes.
