# Supplementary material for: Modeling disordered protein interactions from biophysical principles
Source: PLoS Comput Biol. 2017 Apr 10;13(4):e1005485. doi: 10.1371/journal.pcbi.1005485 (PMC5402988; doi:10.1371/journal.pcbi.1005485)
Supplement: S6 Table — (PDF) [file pcbi.1005485.s016.pdf]

S6 Table: MD-based protein-peptide docking test set

Receptor name	Peptide sequence	Bound PDB ID	chain	L	Unbound PDB ID
Androgen receptor	SSRFESLFAGEKESR	1t7rA	B	15	2am9A
CheY	ASQDQVDDLLDSLGF	2fmfA	B	15	1jbeA
Serine threonine protein kinase PIM1	ARKRRRHPSGPPTA	2c3iB	A	14	2j2iB
Sh3 domain	EGPPPAMPARPT	1sshA	B	12	1ootA
Mineralocorticoid receptor	QQKSLLQQLLTE	2a3iA	B	12	2aa2A
PPAR alpha	ARHKILHRLLQE	2p54A	B	12	1i7gA
AP-2 complex subunit alpha-2	NPKGWVTFEEEE	2vj0A	P	12	1b9kA
Proliferating cell nuclear antigen	KSTQATLERWF	1rxzA	B	11	1rwzA

All protein-peptide test cases with ≥11 amino acid peptides from MD-based protein-peptide docking test sets [18], [19].
