# Supplementary material for: Modeling disordered protein interactions from biophysical principles
Source: PLoS Comput Biol. 2017 Apr 10;13(4):e1005485. doi: 10.1371/journal.pcbi.1005485 (PMC5402988; doi:10.1371/journal.pcbi.1005485)
Supplement: S7 Table — (PDF) [file pcbi.1005485.s017.pdf]

S7 Table: 2-fold cross validation for optimizing Path and Model Scores

Group 1 testing performance					
Bound PDB ID	Recall (%)	RFH	Unbound PDB ID	Recall (%)	RFH
1ycrA	19.8	1	1z1mA	15.2	3
1wkwA	5.3	46	1ipbA	4.1	23
1sb0A	14.1	4	4i9oA	2.7	107
2bzwA	31.1	1	1pq0A	-	-
1devA	1.1	13	1khxA	1.1	7
1jpwA	2.3	2	2z6hA	13.1	8
2c1tA	-	-	1bk5A	-	-

Group 2 testing performance					
Bound PDB ID	Recall (%)	RFH	Unbound PDB ID	Recall (%)	RFH
1fv1AB	10.1	11	4ah2AB	10.1	1
2cpkE	3.3	7	1j3hA	2.4	12
1sqkA	10.3	17	1ijjA	20.3	6
3owtAB	3.5	2	3cz6AB	3.9	49
1p4qB	10.9	9	1l3eB	11.6	2
1l8cA	4.6	93	1u2nA	5.7	15
1xtgA	100.0	1	1xtfA	-	-

Group 1 testing performance shows prediction results using the weights trained on the group 2 complexes, and vice versa. Recall: the number of hits retrieved by the trained score divided by the total number of hits. This is to evaluate the performance of Path Score. RFH: numerical rank of the first hit (good model). This is to evaluate the performance of Model Score. Dash (-) indicates that the complex had no hits.
